# FOXL2 directs DNA double-strand break repair pathways by differentially interacting with Ku

**DOI:** 10.1038/s41467-020-15748-1

**Published:** 2020-04-24

**Authors:** Hanyong Jin, Boeun Lee, Yongyang Luo, Yuri Choi, Eui-Hwan Choi, Hong Jin, Kee-Beom Kim, Sang Beom Seo, Yong-Hak Kim, Hyung Ho Lee, Keun Pil Kim, Kangseok Lee, Jeehyeon Bae

**Affiliations:** 1 Department of Life Science, Chung-Ang University, Seoul, 06974 Korea; 2 School of Pharmacy, Chung-Ang University, Seoul, 06974 Korea; 3 Department of Chemistry, College of Natural Sciences, Seoul National University, Seoul, 08826 Korea; 4 Department of Microbiology, Catholic University of Daegu School of Medicine, Daegu, 42472 Korea

**Keywords:** Acetylation, Non-homologous-end joining

## Abstract

The balance between major DNA double-strand break (DSB) repair pathways is influenced by binding of the Ku complex, a XRCC5/6 heterodimer, to DSB ends, initiating non-homologous end joining (NHEJ) but preventing additional DSB end resection and homologous recombination (HR). However, the key molecular cue for Ku recruitment to DSB sites is unknown. Here, we report that FOXL2, a forkhead family transcriptional factor, directs DSB repair pathway choice by acetylation-dependent binding to Ku. Upon DSB induction, SIRT1 translocates to the nucleus and deacetylates FOXL2 at lysine 124, leading to liberation of XRCC5 and XRCC6 from FOXL2 and formation of the Ku complex. *FOXL2* ablation enhances Ku recruitment to DSB sites, imbalances DSB repair kinetics by accelerating NHEJ and inhibiting HR, and thus leads to catastrophic genomic events. Our study unveils the SIRT1-(de)acetylated FOXL2-Ku axis that governs the balance of DSB repair pathways to maintain genome integrity.

## Abstract (web summary)

The Ku complex, formed by XRCC5/6 heterodimer, binds to double strand break (DSB) ends, initiating non homologous end joining (NHEJ) and preventing homologous recombination (HR). Here, the authors reveal that FOXL2, a forkhead family transcriptional factor, directs DSB repair pathway choice by acetylation-dependent binding to Ku.

## Introduction

Double-strand breaks (DSBs) are highly toxic DNA lesions that are generated by endogenous and exogenous DNA-damaging factors, such as by-products of cellular respiration, replication fork collapse, chemical reagents, and ultraviolet (UV) radiation. Multiple DNA repair pathways play essential roles in maintaining genomic integrity. Failure of DNA lesion repair leads to the accumulation of deleterious mutations, which increases the risk of developing cancer and diverse inherited human diseases^1^. Nonhomologous end joining (NHEJ) and homologous recombination (HR), two representative DSB repair pathways, compete to rejoin DSBs^2^. The choice of DSB repair pathway is critical to balance DNA repair^3,4^. However, how the balance between NHEJ and HR is effectively regulated remains elusive.

NHEJ is a template-independent DSB repair mechanism that is a specialised DBS-end ligation reaction and is active throughout all phases of the cell cycle. NHEJ is rapid, robust, and the primary repair pathway before DNA replication occurs^5,6^. However, it is error-prone and therefore can result in the accumulation of somatic mutations that can cause cancer development and cellular abnormalities. In contrast, HR requires the presence of an intact template DNA, e.g., a sister chromatid or a homologous sequence, and is therefore operational only in the S and G2 phases^5–7^. HR relies on DSB end resection by Mre11-Rad50-NBS1/ExoI, CtIP, and helicase activity, and achieves DNA repair with high fidelity^5^.

In vertebrates, NHEJ is initiated by binding of the Ku complex, a heterodimer of XRCC5 (Ku80) and XRCC6 (Ku70) that serves as a docking site for NHEJ components, to DSB ends, protecting them from nuclease activity^8,9^. The Ku complex acts as a key factor in balancing the DSB repair pathway, along with 53BP1, Rif1, PTIP, and BRCA1, by blocking DSB end resection^2,10^. Ku binds broken DNA ends with strong affinity and recruits DNA-dependent protein kinase catalytic subunit, leading to recruitment of Artemis, XRCC4, DNA ligase IV, and XRCC4-like factor to accomplish NHEJ repair^11,12^. While the molecular processes of NHEJ-based DSB repair after binding of Ku to DSB sites are well characterised, the regulatory network that controls Ku recruitment to DSB sites is unknown.

Forkhead box protein L2 (FOXL2) is a member of the forkhead box (FOX) superfamily of transcriptional factors that possess a DNA-binding domain. Previous studies have found that FOXL2 regulates the transcription of critical genes involved in a wide spectrum of biological processes, including sex determination, steroidogenesis, differentiation, apoptosis, and proliferation^13–19^. FOXL2 is highly expressed in the ovaries and eyelids, and autosomal dominant germline mutations in *FOXL2* cause blepharophimosis, ptosis epicanthus inversus syndrome (BPES) manifested by an eyelid malformation and primary ovarian insufficiency (POI)^20^. More than 97% of ovarian adult-type granulosa cell tumour (AGCT) patients carry an exclusive and prevalent somatic missense mutation (c.402 C > G; p.C134W) in *FOXL2*^21^. FOXL2 undergoes dynamic post-translational modifications that modulate its activity, stability, and subcellular localisation^22–25^. Various FOXL2-binding proteins have been identified. In particular, Veitia and colleagues^26^ reported interaction of ectopically expressed FOXL2 with XRCC6 in COS-7 cells, implying a plausible role of FOXL2 in DBS repair. At present, *FOXL2* mutations are the only genetic factor known to cause BPES and AGCT. However, the pathogenic mechanisms of these *FOXL2* mutations remain unclear.

In the present study, we identify XRCC5 as a FOXL2-binding protein by tandem affinity purification (TAP) of FOXL2-interacting immunoprecipitates followed by LC-MS/MS analysis and investigated the potential role of FOXL2 in DNA damage responses. We show that the status of FOXL2 acetylation controls XRCC5/XRCC6 functional activity and genome integrity by directing DSB repair pathway choice between NHEJ and HR under diverse conditions on DNA damage.

## Results

### FOXL2 interacts with XRCC5/6

**Fig. 1:**
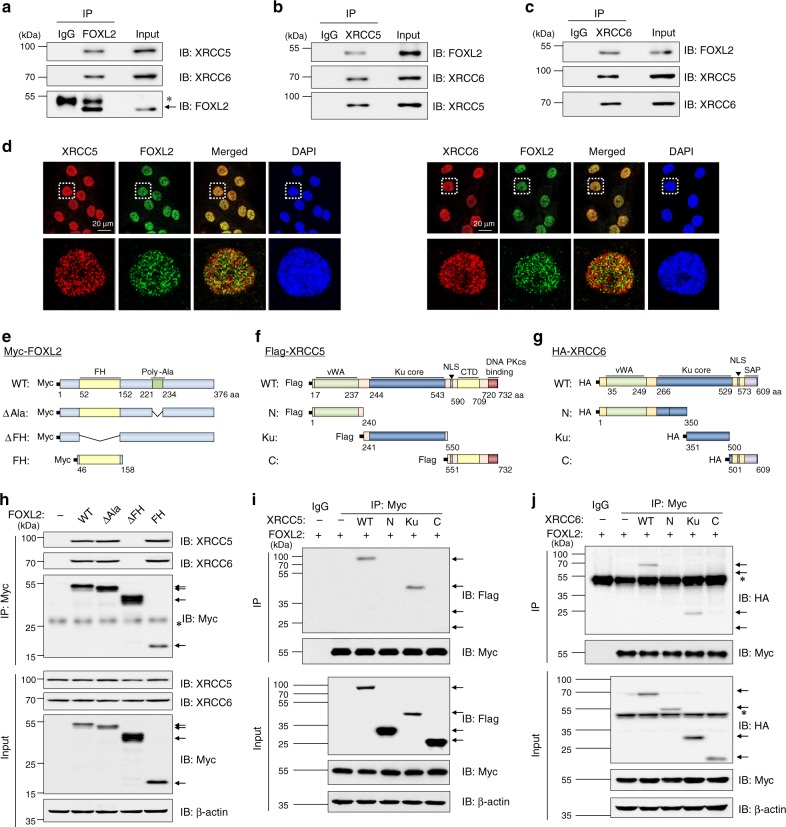
FOXL2 physically interacts with XRCC5 and XRCC6. **a**–**c** Interactions between endogenously expressed FOXL2 and XRCC5 and XRCC6 proteins in KGN cells were demonstrated by immunoprecipitation (IP) with IgG (control), anti-FOXL2 (**a**), anti-XRCC5 (**b**), or anti-XRCC6 (**c**) antibody followed by immunoblot analysis with the indicated antibodies. **d** Nuclear colocalisation of endogenous FOXL2 protein with XRCC5 and XRCC6 in KGN cells revealed by confocal fluorescence microscopy. The length of scale bar is 20 μm. **e**–**g** Schematic representations of the plasmids encoding full-length (WT) and truncated mutants of FOXL2 (**e**), XRCC5 (**f**), and XRCC6 (**g**), which were generated to determine the binding domains. FH: forkhead domain; poly-Ala: polyalanine region; vWA: von Willebrand A domain; NLS: nuclear localisation signal; CTD: C-terminal helical domain; SAP: SAF-A/B, Acinus and PIAS domain. **h**–**j** 293T cells were transfected with the indicated plasmids. Cell lysates were subjected to IP with control IgG or anti-Myc antibodies followed by immunoblotting with the indicated antibodies. The arrows indicate expected positions of the respective proteins, and nonspecific bands are indicated with asterisks in all panels. Source data are provided as a Source Data file.

FOXL2 interacts with various transcription factors and DNA damage repair proteins, including XRCC6 and SIRT1 deacetylase^26^. Here, we explored the FOXL2 interactome further by immunoprecipitation (IP) using calmodulin-streptavidin-tagged FOXL2. 293T cells were transfected with plasmid encoding calmodulin-streptavidin-tagged FOXL2 or empty plasmid. Immunoprecipitates were subjected to TAP and LC-MS/MS analysis. The FOXL2 and its interacting nuclear proteins were identified based on the tandem affinity purification and gel-based mass spectrometry (Supplementary Fig. 1). LC-MS/MS was applied to analyse 21 candidate proteins in SDS-PAGE analysis with the TAP samples of tagged FOXL2. From the mass spectrometry results scored by X!Tandem, we finally identified 5 potential FOXL2-interacting proteins including XRCC5, by filtering for the highest match score (corrected %) and lowest log(e) value (Supplementary Table 1). The physical interaction of XRCC5 with FOXL2 overexpressed in 293T cells was confirmed by IP analysis (Supplementary Fig. 2a). As FOXL2 is highly expressed in ovarian granulosa cells and plays pivotal roles in ovarian development^14,15^, the interaction of FOXL2 with XRCC5 and XRCC6 was examined in human ovarian granulosa cell tumour-derived KGN cells. Endogenous FOXL2 interacted with both XRCC5 and XRCC6 in KGN cells (Fig. 1a–c). In addition, the physical interaction between endogenous FOXL2 and XRCC5 was also observed in various non-tumour cell lines (Supplementary Fig. 2b–d). Immunofluorescence analysis showed that XRCC5/66 colocalise with FOXL2 in the nucleus (Fig. 1d).

### The forkhead domain interacts with the Ku core domain

FOXL2 contains a forkhead DNA-binding domain (FH) required for DNA binding and a polyalanine stretch of fourteen residues that presumably functions in proper folding of FOXL2^27^. Expression constructs encoding Myc-tagged full-length wild-type (WT) FOXL2, polyalanine-deleted FOXL2 (ΔAla), forkhead domain-deleted FOXL2 (ΔFH), and forkhead domain of FOXL2 (FH) (Fig. 1e), were generated and used for IP with an anti-Myc antibody in 293T cells. All FOXL2 forms, except the ΔFH mutant, showed binding ability to both XRCC5 and XRCC6 (Fig. 1h). The FH domain itself was sufficient for interaction with XRCC5/6, implying that these Ku proteins bind to the FH domain of FOXL2 (Fig. 1h).

To determine the FOXL2-interaction domain of XRCC5, we generated mutant constructs, including XRCC5-N (amino acids [aa] 1–240), XRCC5-Ku (aa 241–550), and XRCC5-C (aa 551–732), each fused with a Flag tag (Fig. 1f). Co-IP revealed that XRCC5-Ku, but not XRCC5-N and XRCC5-C, physically interacted with FOXL2, indicating that the Ku core domain of XRCC5 interacts with FOXL2 (Fig. 1i). The FOXL2-binding domain of XRCC6 was determined using XRCC6-N (aa 1–350), XRCC6-Ku (aa 351–500), and XRCC6-C (aa 501–609) mutants (Fig. 1g). Again, the Ku core domain was sufficient for interaction with FOXL2 (Fig. 1j).

### FOXL2 competitively inhibits Ku heterodimer formation

**Fig. 2:**
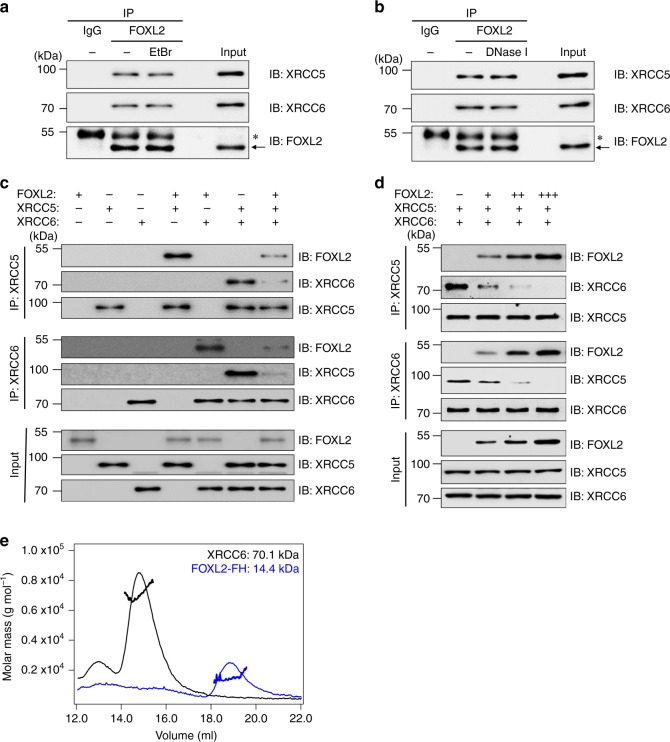
FOXL2 inhibits Ku heterodimer formation. **a**, **b** To determine whether ternary complex formation between FOXL2 and Ku proteins requires the presence of DNA, ethidium bromide (EtBr, 50 μg/ml) (**a**) or DNase I (10 U) (**b**) were added to KGN lysates, which were subjected to IP using anti-FOXL2 antibody followed by western blotting. **c**, **d** In vitro complex formation of FOXL2 with Ku proteins was assessed using purified recombinant His-tagged FOXL2, XRCC5, and XRCC6 proteins. Following incubation of the indicated proteins (0.3 μg) (**c**), IP with anti-XRCC5 or anti-XRCC6 antibody and immunoblot analyses with the indicated antibodies were conducted. In addition, increasing amounts (0.15, 0.3, and 0.6 μg) of FOXL2 protein (**d**) were incubated with equal amounts (0.3 μg) of XRCC5 and XRCC6 proteins, and IP and immunoblot analyses were conducted. **e** Monomeric existence of XRCC6 (black line) and FOXL2-FH (blue line) was analysed by SEC-MALS. The thick lines represent measured molecular mass. The expected theoretical molecular mass of monomeric XRCC6 and FOXL2-FH are 70.9 kDa and 13.7 kDa, respectively. Source data are provided as a Source Data file.

As the Ku heterodimer binds avidly to DSB ends^28^ and FOXL2 binds DNA via its FH domain^29^, we assessed whether association of FOXL2 with XRCC5/6 depends on the presence of DNA. Ethidium bromide (EtBr), which selectively disrupts protein–DNA interactions^30^, or DNase I, a nonspecific endonuclease that induces DNA cleavage^31^, was added to KGN lysate to eliminate the possibility of nonspecific DNA binding. FOXL2 retained binding capacity to XRCC5/6 even after the addition of EtBr or DNase I (Fig. 2a, b), indicating that the interaction of FOXL2 with XRCC5/6 does not require the presence of DNA.

To examine the role of FOXL2 in the binding interaction between XRCC5 and XRCC6, we conducted an in vitro binding assay using recombinant His-tagged FOXL2, XRCC5, and XRCC6 (Supplementary Fig. 3a, b). In the absence of DNA, XRCC5 and XRCC6 were both capable of binding FOXL2 in vitro (Fig. 2c). However, FOXL2 dramatically suppressed complex formation between XRCC5 and XRCC6 (Fig. 2c, d). We found that purified XRCC5 protein was easily aggregated, which renders XRCC5 insufficiently stable to further analyse binding characteristics, while XRCC6 was stable. Thus, we used XRCC6 (aa 1-609) and the FH domain of FOXL2 (aa 46–158) for further analyses of their structural interaction. Because FOXL2 competitively interacts with XRCC5 and XRCC6 and XRCC5/6 complex forms 1:1 heterodimer, we reasoned that XRCC6 alone might exist as a monomer in solution and form a 1:1 heterodimer with FOXL2. Indeed, based on size-exclusion chromatography with multi-angle light-scattering (SEC-MALS) data, XRCC6 and the FH domain of FOXL2 themselves exist as a monomer in solution (Fig. 2e), although a direct demonstration of a 1:1 heterodimer formation between FOXL2 and XRCC6 could not be done in this setting. These data indicate that FOXL2, XRCC5, and XRCC6 competitively interact with each other and that FOXL2 inhibits XRCC5/6 heterodimer formation.

### FOXL2 inhibits Ku-mediated NHEJ, but stimulates HR

**Fig. 3:**
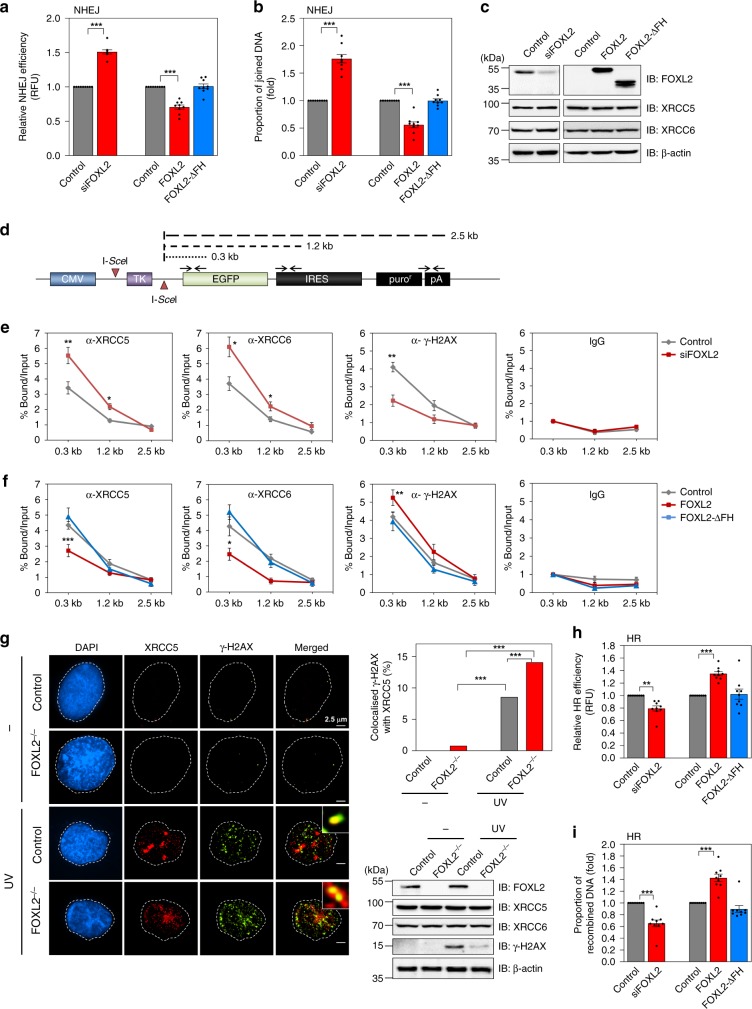
FOXL2 inhibits Ku-mediated DNA double-strand break (DSB) repair. **a**, **b** The effect of FOXL2 on DSB repair was assessed by reporter-based quantification of NHEJ repair in 293TdA3-stable cells cotransfected with an I-*Sce*I endonuclease expression plasmid. The EGFP-positive cell fraction (RFU; relative fluorescence unit) (**a**) and the proportion of nonhomologous end-joined DNA (**b**) were determined in 293TdA3-stable cells cotransfected with an I-*Sce*I endonuclease expression plasmid along with control or FOXL2 siRNA, or plasmid expressing empty vector (control), FOXL2, or FOXL2-ΔFH. **c** Immunoblot results of 293TdA3-stable cells after transfection with the indicated siRNAs or plasmids are shown on the right panel. **d** Illustration of the target sites of primers used for quantitative PCR analysis of chromatin immunoprecipitates of 293TdA3 cells expressing I-*Sce*I presented in **e** and **f**. **e**, **f** ChIP was performed with antibodies against XRCC5, XRCC6, γ-H2AX, or control IgG after transfection of control or FOXL2 siRNA (**e**) or plasmid expressing empty vector (control), FOXL2, or FOXL2-ΔFH (**f**). **g** The influence of FOXL2 on XRCC5 localisation to DNA damage foci was analysed by immunocytochemistry. FOXL2-knockout (FOXL2^–/–^) and control KGN cells were irradiated with UV (100 J/m^2^) for 2 h. The colocalisation percentage of γ-H2AX with XRCC5 is shown in the upper right panel. For each group, at least 40 randomly selected cells from three independent slides were analysed. The length of scale bar is 2.5 μm. Immunoblot analysis of expression levels of proteins, including γ-H2AX, is presented in the lower right panel. **h**, **i** The effect of FOXL2 on homologous recombination (HR) DNA repair was assessed by reporter-based quantification of HR repair. The GFP-positive cell fractions (**h**) and recombined DNA (**i**) were quantified in 293T-DR-GFP stable cells transfected with an I-*Sce*I endonuclease expression plasmid along with siRNA for control or FOXL2, or plasmid expressing empty vector (control), FOXL2, or FOXL2-ΔFH. Data are presented as the mean ± SEM of three independent experiments performed in triplicate. The *p* values were analysed by unpaired, two-tailed Student’s *t* test (*p < 0.05, **p < 0.01, ***p < 0.001). Source data are provided as a Source Data file.

Binding of XRCC5/6 to DNA break ends is required to initiate NHEJ-mediated DSB repair^11^, and the data above indicate that FOXL2 attenuates Ku complex formation. Thus, we surmised that FOXL2 can directly inhibit Ku-mediated NHEJ. To assess this hypothesis, we used 293TdA3 cells harbouring IRES-TK-EGFP plasmid containing two recognition sites for I-*Sce*I endonuclease stably integrated into the genomic DNA, which has been proven to be an effective system to assess DSB repair by NHEJ^32^. When FOXL2 was depleted in the 293TdA3 cells, NHEJ activity was significantly increased based on measurements of both EGFP-positive cells and the proportion of joined DNA in cells transfected with an I-*Sce*I expression plasmid (Fig. 3a, b). Accordingly, overexpression of FOXL2 decreased the NHEJ efficiency, whereas the FH-deleted mutant (ΔFH), which lacks XRCC5- and XRCC6-binding capacity, did not have altered NHEJ efficiency (Fig. 3a, b). FOXL2 depletion or overexpression did not affect the expression levels of XRCC5/6, indicating that NHEJ inhibition by FOXL2 is not due to changes in XRCC5/6 expression (Fig. 3c). In addition, we confirmed that FOXL2 does not induce DNA damage per se (Supplementary Fig. 4a–c).

We determined the role of FOXL2 in XRCC5/6 recruitment to DSB sites by chromatin immunoprecipitation (ChIP) analysis in 293TdA3 cells. DSBs were induced by I-*Sce*I digestion in cells transfected with control siRNA or siFOXL2. Then, ChIP was performed with antibody against immunoglobulin G (IgG), XRCC5, XRCC6, or serine 139-phosphorylated H2AX (γ-H2AX), an early DSB marker^33^. The immunoprecipitates were subjected to quantitative PCR (qPCR) using primers covering three regions of pIRES-TK-EGFP (0.3, 1.2, and 2.5 kb)^32^, as indicated in Fig. 3d. FOXL2 knockdown significantly increased the enriched DNA fragments in XRCC5 and XRCC6 immunoprecipitates and decreased those in the γ-H2AX immunoprecipitate, especially in the 0.3-kb stretch from the I-*Sce*I site (Fig. 3e). Conversely, upon FOXL2 overexpression, enriched DNA fragments in XRCC5 or XRCC6 immunoprecipitates were reduced, whereas they were increased in γ-H2AX immunoprecipitate (Fig. 3f). In contrast to WT, the null mutant of FOXL2 (ΔFH) failed to modulate the recruitment of XRCC5, XRCC6, or γ-H2AX to DSB regions (Fig. 3f).

Next, we generated a FOXL2-knockout (KO) KGN cell line using a CRISPR/Cas9-nickase-based system (Supplementary Fig. 5a, b), which has minimal off-target events^34^. Sequencing analysis indicated that the FOXL2 KO cell line expresses fragments of FOXL2 that lack the critical FH domain (Supplementary Fig. 5b). Using this cell line, we performed immunofluorescence analysis of XRCC5 and γ-H2AX to confirm that FOXL2 depletion increases XRCC5 accumulation at DSB sites. Cells were treated with cytoskeleton (CSK) buffer supplemented with RNase A, which allows specific detection of DSB-bound proteins^35^. Without DNA damage insult, colocalisation of XRCC5 with γ-H2AX was negligible in control and FOXL2-KO cells (Fig. 3g). Upon UV irradiation, colocalisation of XRCC5 and γ-H2AX foci was increased in control cells, and even more so in FOXL2-KO cells (Fig. 3g). Western blot analysis of UV-exposed control and FOXL2-KO cells revealed that the γ-H2AX levels were dramatically decreased in the KO cells (Fig. 3g), which likely reflects enhanced Ku recruitment to DSB sites and DSB repair by FOXL2 depletion. Unlike in control KGN cells, the γ-H2AX levels was not significantly altered in FOXL2-KO cells at different times after UV light exposure, which may reflect faster DSB repair in cells lacking FOXL2 (Supplementary Fig. 6). Together, these data indicated that FOXL2 inhibits NHEJ-mediated DSB repair by sequestering Ku complex away from DNA lesion sites.

As Ku-mediated NHEJ and HR compete for DSB repair through interference of Ku with HR initiation^36,37^, we investigated the influence of FOXL2 on the HR pathway using 293T cells harbouring pDR-GFP containing an I-*Sce*I recognition site^38^. FOXL2 depletion and overexpression significantly reduced and enhanced HR activity, respectively, based on assessment of both GFP-positive cells and the proportion of recombined DNA in cells transfected with an I-*Sce*I expression plasmid (Fig. 3h, i). In contrast, HR activity was not affected by ectopic expression of the null mutant (ΔFH) of FOXL2 (Fig. 3h, i). These effects were exact the opposite of those observed for NHEJ upon FOXL2 modulation (Fig. 3a, b), implying that the inhibitory action of FOXL2 on Ku-mediated NHEJ may lead to HR stimulation.

### DSBs induce nuclear translocation of SIRT1 and Ku release

**Fig. 4:**
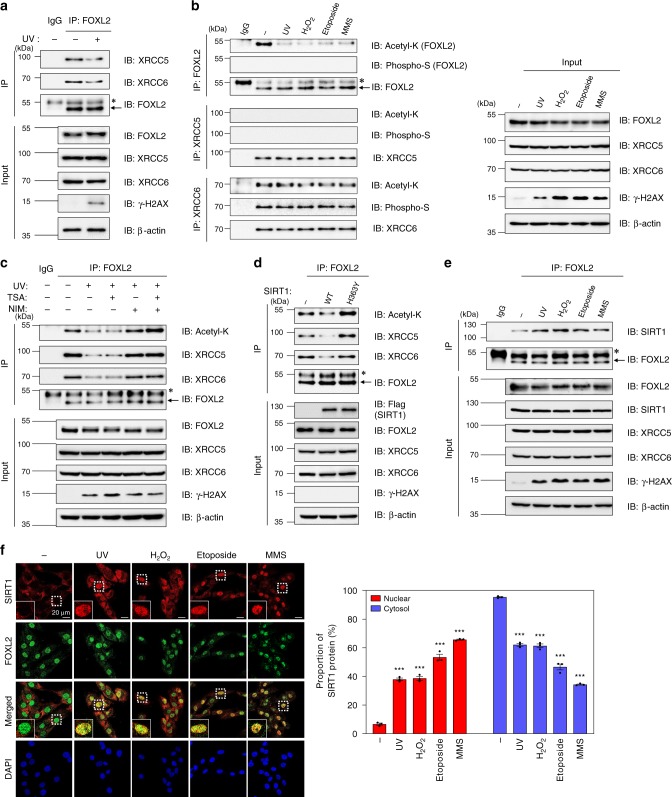
XRCC5 and XRCC6 are released from FOXL2 in response to DSB stresses via deacetylation of FOXL2 by SIRT1. **a** The effect of UV damage on ternary complex formation of FOXL2 with XRCC5/XRCC6 was determined by IP with anti-FOXL2 antibody and immunoblot analyses in KGN cells. **b** Changes in lysine acetylation and serine phosphorylation of FOXL2, XRCC5, and XRCC6 proteins upon DSB insults were assessed following exposure of KGN cell to UV (100 J/m^2^, 2 h), H_2_O_2_ (150 µM, 2 h), etoposide (100 µM, 1 h), or methyl methanesulfonate (MMS; 1 mM, 30 min). The cell lysates were immunoprecipitated with anti-FOXL2, -XRCC5, or -XRCC6 antibodies. The immunoprecipitates were immunoblotted using anti-acetylated lysine (Acetyl-K) and anti-phosphorylated serine (pSer) antibodies. **c** KGN cells were treated with trichostatin A (TSA; 1 µM) or nicotinamide (NIM; 5 mM) after UV exposure, and changes in FOXL2 acetylation and FOXL2 interaction with XRCC5/XRCC6 were analysed by IP with anti-FOXL2 antibody. **d** KGN cells were transfected with plasmids encoding Flag-tagged wild-type SIRT1 (WT) or non-catalytic mutant SIRT1 (H363Y). The cell lysates were immunoprecipitated with anti-FOXL2 antibody following immunoblot analyses using the indicated antibodies. **e** Changes in FOXL2-interaction with SIRT1 after DSB insults were assessed by IP followed by immune blotting of KGN cell lysates. **f** DSB insult-induced changes in colocalisation of FOXL2 with SIRT1 in KGN cells were determined by confocal microscopy using anti-FOXL2 and -SIRT1 antibodies. The length of scale bar is 20 μm. The proportions of nuclear and cytosolic SIRT1 were quantified using Zen software and presented in the right panel. Data are presented as the mean ± SEM of three independent experiments performed in triplicate. The *p* values were analysed by unpaired, two-tailed Student’s *t* test (***p < 0.001). Source data are provided as a Source Data file.

Next, we examined the molecular interaction between FOXL2 and XRCC5/6 following DSB induction by UV irradiation, H_2_O_2_, and cytotoxic agents, including etoposide, and methyl methanesulfonate (MMS). Upon UV exposure, interaction of FOXL2 with both XRCC5 and XRCC6 was significantly diminished in KGN cells (Fig. 4a), and this effect was consistently observed in other types of cancer cells (Supplementary Fig. 7a–c). This weakened interaction was not due to decreased expression of FOXL2, XRCC5, or XRCC6 (Fig. 4a and Supplementary Fig. 7a–c), nor due to changes in their subcellular localisation following DNA DSBs (Supplementary Fig. 8a–d). Similarly, H_2_O_2_, etoposide, and MMS decreased FOXL2 complex formation with XRCC5/6 (Supplementary Fig. 9a–c).

Of interest, the acetylated form of FOXL2 was prominently decreased following DSB induction, whereas serine phosphorylation of FOXL2 was unaffected (Fig. 4b). Therefore, we investigated whether FOXL2 acetylation affects its affinity for XRCC5/6. KGN cells were exposed to UV and treated with trichostatin A (TSA), an inhibitor of histone deacetylase (HDAC) I/II, or nicotinamide (NIM), an inhibitor of SIRT1 deacetylase, and cell lysates were immunoprecipitated with anti-FOXL2 antibody. The UV-induced decrease in FOXL2–XRCC5/6 interaction was dramatically restored by treatment with NIM, but not TSA (Fig. 4c). Concurrently, the suppression of FOXL2 acetylation upon UV irradiation was completely restored by treatment with NIM, but not TSA (Fig. 4c), indicating that SIRT1 mediates FOXL2 deacetylation. Overexpression of SIRT1 led to a decrease in the level of acetylated FOXL2, whereas overexpression of a null mutant of SIRT1 (H363Y) did not (Fig. 4d). Concurrently, FOXL2–XRCC5/6 complex formation was dramatically diminished upon SIRT1 overexpression, but not upon overexpression of the H363Y mutant (Fig. 4d). In contrast, when SIRT1 was depleted under the UV-induced DNA damage condition, the deacetylation of FOXL2 was clearly prevented and FOXL2 interactions with XRCC5/6 were weakened (Supplementary Fig. 10), which is analogous effects observed by SIRT1 inactivation (Fig. 4c, d). These results imply that XRCC5/6 preferentially bind to acetylated FOXL2.

Further, we found that FOXL2–SIRT1 interaction was increased following DSB insults, while the SIRT1 level remained unchanged (Fig. 4e). To elucidate the mechanism underlying the increased association of the two proteins, we examined changes in the intracellular localisation of SIRT1 following DNA damage. We observed that endogenous SIRT1 was mainly present in the cytoplasm of KGN cells as determined by confocal microscopy (Fig. 4f) and immunoblot analysis of subcellular fractionated cell lysates (Supplementary Fig. 11). In sharp contrast, DSB stresses induced significant nuclear translocation of cytoplasmic SIRT1, leading to increased nuclear colocalisation of SIRT1 and FOXL2 (Fig. 4f and Supplementary Fig. 11).

### Acetylation of the K124 of FOXL2 prevents NHEJ repair

**Fig. 5:**
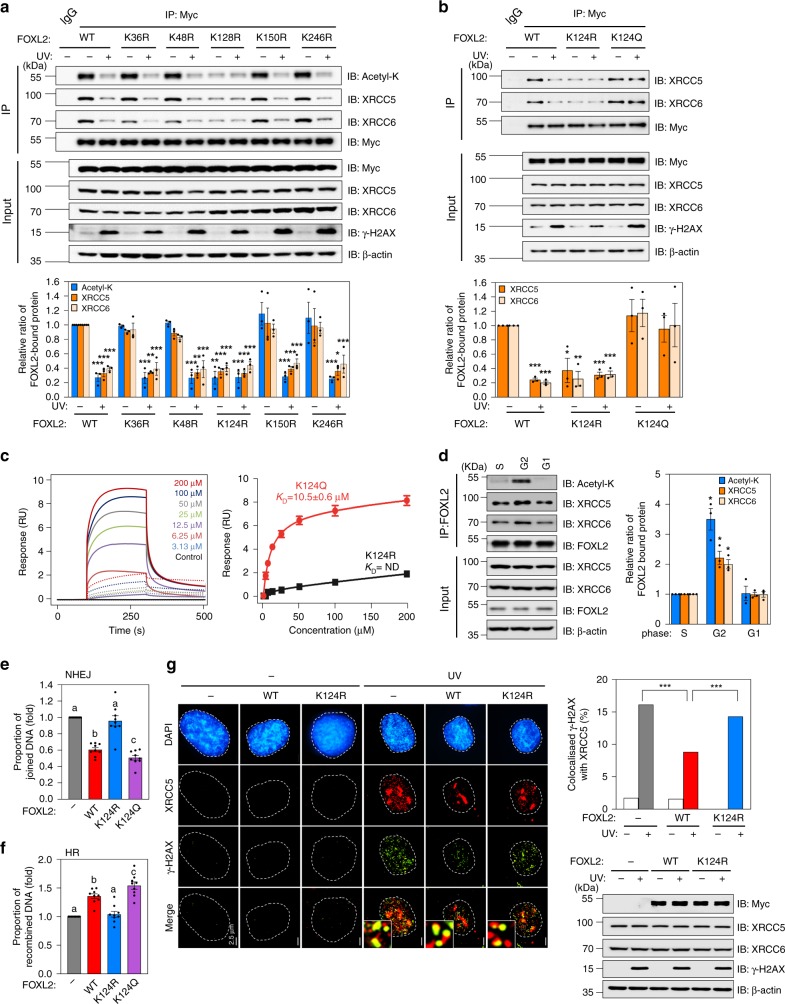
DSB stress leads to deacetylation of K124 of FOXL2, which results in Ku liberation from FOXL2 followed by Ku localisation to DSB sites. **a** KGN cells were transfected with plasmids encoding Myc-FOXL2 or five lysine mutants of FOXL2. Following UV irradiation, cell lysates were immunoprecipitated with anti-Myc antibody and analysed by immunoblotting (upper panel). The immunoblot results of acetylated FOXL2 and FOXL2-bound XRCC5/XRCC6 were quantified with Quantity One software (Bio-Rad Laboratories) and normalised to that of Myc-FOXL2 (lower panel). **b** KGN cells overexpressing WT or the K124R or K124Q were UV-exposed. Representative immunoblots (upper panel) and quantitative data (lower panel) are presented. **c** Representative BLI assays of XRCC6 and the FH domain of FOXL2 (K124Q and K124R) are shown. BLI sensorgrams (K124Q and K124R in solid and dotted lines, respectively) with different concentrations of analytes are indicated by different colours. Equilibrium analysis and calculated *K*_D_ are shown at right panel. **d** Cell cycle-synchronised KGN cells (Supplementary Fig. 9) were incubated with 1 mM MMS for 30 min to induce DSBs. The cell lysates were immunoprecipitated with anti-FOXL2 antibody and analysed by immunoblotting (left upper panel) and quantified (right panel). **e**, **f** The NHEJ (**e**) and HR (**f**) activities of WT, K124R, and K124Q were assessed in 293TdA3- or 293T-DR-GFP stable cells expressing I-*Sce*I and are presented as joined DNA (**e**) or recombined DNA (**f**), respectively. Data are the mean ± SEM of three independent experiments performed in triplicate. Different letters denote statistical significance (Student–Newman–Keuls test; *p* < 0.05). **g** The influence of WT and K124R on the localisation of XRCC5 to DNA damage foci was analysed by immunocytochemistry (left panel). The length of scale bar is 2.5 μm. The colocalisation percentage of γ-H2AX with XRCC5 is also shown (upper right panel). For each group, at least 40 randomly selected cells from three independent slides were analysed. Data are the mean ± SEM of three independent experiments, and statistically significant differences were analysed by unpaired, two-tailed Student’s *t* test (*p < 0.05, **p < 0.01, ***p < 0.001). Source data are provided as a Source Data file.

To predict acetylated residue(s) of FOXL2, we analysed its protein sequence using Prediction of Acetylation on Internal Lysines (http://bdmpail.biocuckoo.org/prediction.php) and Acetylation Set Enrichment-Based (http://bioinfo.bjmu.edu.cn/huac/predict_p/). Five lysine (K) residues of FOXL2, i.e., K36, K48, K124, K150, and K246, of which K48, K124, K150, and K246 are evolutionarily well conserved across diverse phyla (Supplementary Fig. 12), yielded high predictive scores by both tools. We generated acetylation-resistant FOXL2 mutants in which the above five lysine residues were replaced with arginine (R), and we determined changes in their acetylation status upon UV irradiation. The K124R mutant exhibited a significantly lower steady-state acetylation level than the WT and other FOXL2 mutants (Fig. 5a). The acetylation levels of WT FOXL2 and the FOXL2 mutants, except K124R, were significantly decreased following UV exposure (Fig. 5a). Unlike the other FOXL2 mutants, the K124R mutant exhibited very weak association with both XRCC5 and XRCC6, and this weak complex formation was not affected by UV irradiation (Fig. 5a). We also generated and tested a K124Q mutant of FOXL2, in which K124 was substituted with glutamine (Q), to mimic a constitutively acetylated state. In striking contrast to K124R, the K124Q mutant showed interaction affinity to XRCC5/6 similar to that of the WT, and its interaction with the Ku complex was not significantly altered after UV exposure (Fig. 5b). To further confirm the importance of acetylation status of K124 of FOXL2 for XRCC6 interaction, the binding affinities of K124Q or K124R mutated FH domain (aa 46–158) to XRCC6 (aa 1-609) were examined by a bio-layer interferometry (BLI) experiment. The K124Q mutated FH domain bound to the XRCC6 with a dissociation constant (*K*_D_) of 10.5 ± 0.6 μM (Fig. 5c). In contrast, consistent with the above IP results, the K124R mutation of the FH domain nearly abolished binding to XRCC6 in our BLI experiment (Fig. 5c).

In addition, we determined whether FOXL2 acetylation and its association to XRCC5/6 are cell cycle-dependent event. Following MMS-induced DSB, cell cycles of KGN cells were synchronised using double thymidine block and analysed 0, 4, 9, 14 h after the release, in which G1, S, G2, and G1, respectively, were predominant cell cycle phages (Supplementary Fig. 13). The amount of acetylated FOXL2 was dramatically increased with concurrent increase of XRCC5/6-bound to FOXL2 during G2 phage (Fig. 5d). This result implies that the presence of cell cycle–dependent FOXL2-mediated NHEJ regulatory mechanism where sequestering of XRCC5/6 by acetylated FOXL2 during G2 allows HR to occur preferentially over NHEJ.

Furthermore, we investigated whether the acetylation status of FOXL2 at K124 indeed modulates NHEJ pathway. As shown in Fig. 5e and Supplementary Fig. 14a, the acetylation-resistant K124R mutant failed to effectively inhibit NHEJ when compared to the WT FOXL2, whereas the acetylation-mimicking K124Q mutant efficiently inhibited NHEJ-mediated repair activity in 293TdA3 cells. This K124R mutant also failed to modulate HR activity and the K124Q mutant significantly increased HR-mediated DNA repair activity compared to the WT FOXL2 in 293TDR cells (Fig. 5f and Supplementary Fig. 14b). The fractions of uncut DNA were similar among cells expressing WT, K124R, or K124Q FOXL2 (Supplementary Fig. 14c). To confirm the critical role of K124 acetylation of FOXL2 in controlling Ku recruitment to DSB sites, we knocked in WT and K124R into FOXL2-KO cells. Knock-in of WT FOXL2 dramatically decreased the degree of XRCC5 recruitment to γ-H2AX foci by 50% of that in control KGN cells following UV exposure (Fig. 5g). In contrast, knock-in of K124R, which exhibits much weaker binding affinity to XRCC5/6, resulted in a similar degree of XRCC5 localisation to γ-H2AX foci upon UV exposure as that observed in FOXL2 KO cells (Fig. 5g). Together, these results indicated that acetylation of FOXL2 at K124 is a crucial event that allows XRCC5/6 sequestration away from DSB sites and thus prevents NHEJ-mediated DSB repair.

### FOXL2 is required for maintaining genome integrity

**Fig. 6:**
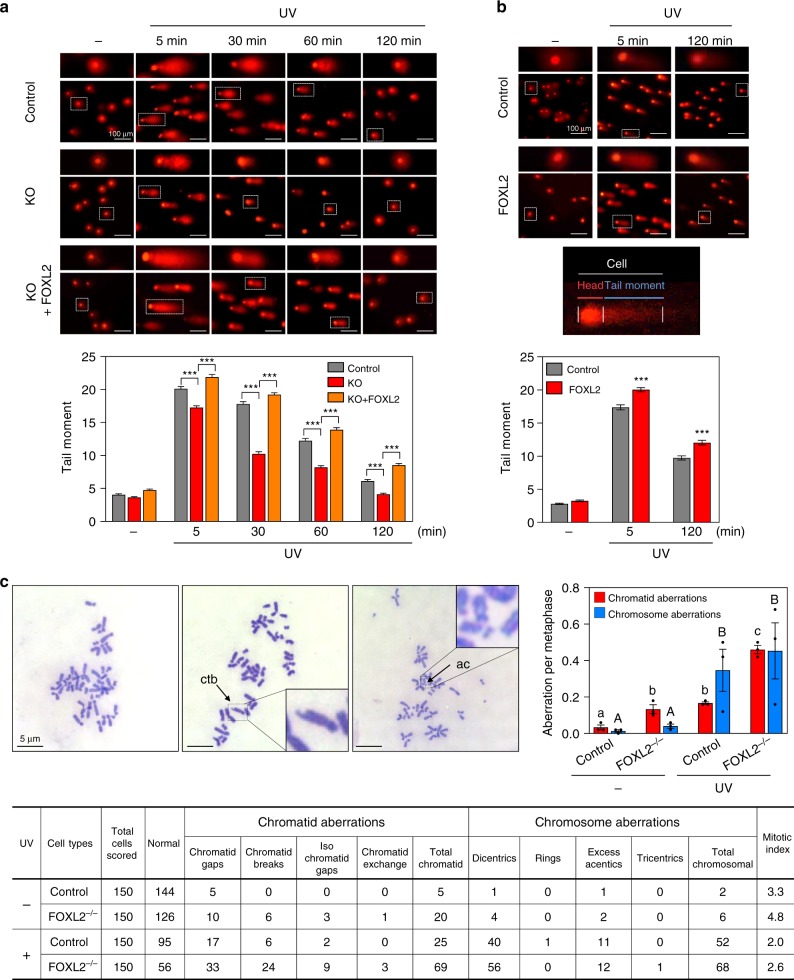
FOXL2 is necessary for maintaining genome stability. **a**, **b** The effect of FOXL2 on UV-induced DBS repair kinetics was analysed by a neutral comet assay. Control (WT), FOXL2-knockout (FOXL2^−/−^), and FOXL2-knocked-in FOXL2^−/−^ (FOXL2^−/−^ + FOXL2) cells of KGN (**a**) along with control and FOXL2-overexpressing KGN cells (**b**) were exposed to UV and their tail moments were measured at the indicated time points. Representative comet images are shown on the top (scale bar, 100 µm). Tail moments were quantified in 120 comets (**a**) and 150 comets (**b**) from two independent experiments using CaspLab software (lower panel). The *p* values were analysed by unpaired, two-tailed Student’s *t* test (***p < 0.001). **c** The influence of FOXL2 on genome integrity was determined by chromosomal aberration analysis in WT and FOXL2^−/−^ KGN cells. At 21 h after UV irradiation, the cells were incubated with colcemid for 3 h, and the frequencies of chromosomal and chromatid aberrations in first metaphase were analysed. Representative Giemsa-stained metaphases from the cells (left upper) and quantitative data (right upper) are shown. The length of scale bar is 5 µm. Numerical data are provided in the table (lower panel). Aberrations are indicated by arrows; ctb, chromatid break; ac, excess acentric fragment. Data are presented as mean ± SEM of three independent experiments. Different letters denote statistical significance (Student–Newman–Keuls test; *p* < 0.05). Source data are provided as a Source Data file.

To evaluate the influence of FOXL2 on DNA repair kinetics further, we used a neutral comet-based assay. The length of the comet tail moment, which represents the degree of DNA breaks, was increased in control KGN cells after UV irradiation (Fig. 6a). In contrast, FOXL2 KO cells exhibited significantly shorter tail moments (Fig. 6a). When FOXL2 was knocked-in to the FOXL2^−/−^ cells, lengths of the comet tail moment were significantly increased (Fig. 6a). Furthermore, we also examined comet assays following ectopic expression of FOXL2 in control KGN cells and observed consistent longer tail moments in cells overexpressing FOXL2 compared to empty vector-transfected cells (Fig. 6b). Tail moment reduction was also observed upon FOXL2 knockdown in KGN cells (Supplementary Fig. 15a).

As DSBs pose a serious threat to genome integrity and cell growth, improper DSB repair systems can cause chromosomal aberrations or genome instability^39^. Thus, we further examined chromosome abnormalities by assessing chromosome spreads at the first metaphase after UV irradiation. The extent of chromosomal aberrations was exacerbated in both FOXL2-KO and -knockdown KGN cells (Fig. 6c and Supplementary Fig. 15b). Especially, the incidence of chromatid-type aberrations was increased by 3–4 folds in FOXL2-depleted cells, whereas the incidence of chromosomal aberrations was not significantly altered in FOXL2-depleted cells (Fig. 6c and Supplementary Fig. 15b). Taken together, we concluded that these chromatid aberrations may reflect the positive role of FOXL2 in HR repair (Fig. 3h, i).

### Pathogenic FOXL2 mutants are defective in NHEJ and HR

**Fig. 7:**
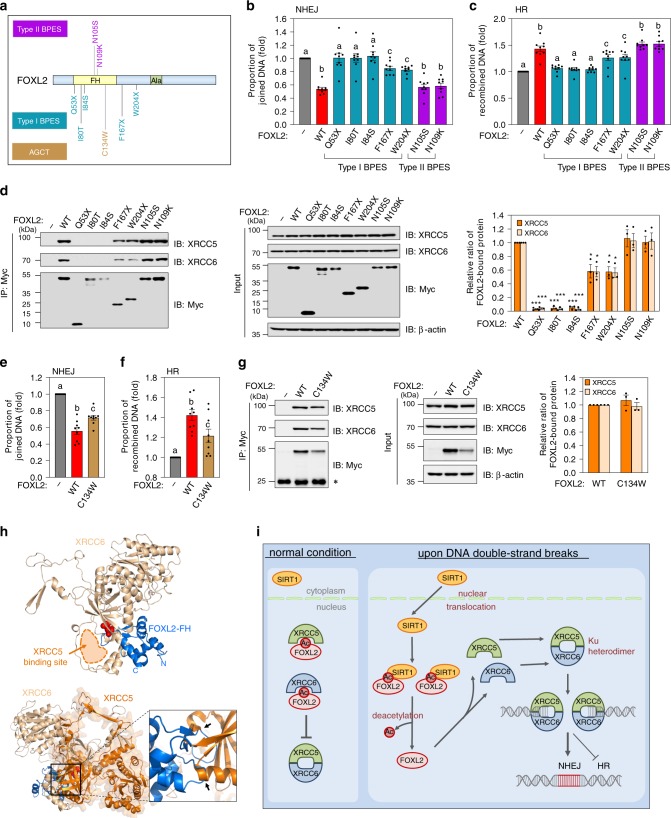
Pathogenic mutants of FOXL2 are associated with defective NHEJ and HR repairs. **a** Schematic representation of BPES- and AGCT-associated mutation sites in FOXL2 is shown. **b**, **c** The effects of BPES-associated pathogenic FOXL2 mutants on NHEJ repair were examined. The proportion of nonhomologous end-joined (**b**) and recombined DNA (**c**) were analysed. **d** 293T cells were transfected with pCMV-Myc vector, WT and mutated FOXL2s, and the lysates were immunoprecipitated with anti-Myc antibody. Binding fractions of XRCC5/6 were detected by immunoblotting (left). Band intensities of immunoprecipitated XRCC5/6 were quantified following normalisation using pull-downed FOXL2 (right). **e**, **f** The effect of AGCT-associated pathogenic FOXL2 mutant (C134W) on NHEJ (**e**) and HR (**f**) repair was examined. The proportion of nonhomologous end-joined (**e**) and recombined DNA (**f**) were analysed. **g** 293T cells were transfected with Myc-tagged WT or C134W FOXL2. IP (left) and quantitative data (right) generated as described in **d**. Data are presented as mean ± SEM of three independent experiments. Different letters denote statistical significance (Student–Newman–Keuls test; *p* < 0.05). Asterisks indicate statistically significant values as analysed by unpaired, two-tailed Student’s *t* test (*p < 0.05, ***p < 0.001). Source data are provided as a Source Data file. **h** Integrative docking model of the FOXL2-XRCC6 complex is presented. Acetylation-mimic Gln124 is highlighted in red. FOXL2-FH, XRCC5, and XRCC6 are coloured in marine, orange, and light orange, respectively. The occupied site of XRCC5 is shown with orange dashed line, and steric clashes between XRCC5 and FOXL2 are shown by black arrows. **i** Pathways of DSB repair highlight**i**ng how post-translational modification by FOXL2 regulates the XRCC5/6 complex formation as part of the NHEJ pathway. The proposed signalling cascade of the NHEJ repair pathway, in which FOXL2 plays a central role is shown. Under normal conditions, FOXL2 acetylated at K124 readily binds to both XRCC5 and XRCC6 and thus inhibits Ku complex formation. However, upon DSB induction, nuclear translocation of SIRT1 increases, leading to K124 deacetylation. As deacetylated FOXL2 has negligible binding affinity to XRCC5/6, XRCC5/6 liberated from FOXL2 readily form heterodimers that bind to DSB lesions and initiate NHEJ repair.

We selected and cloned the following FOXL2 mutants found in BPES patients based on the human FOXL2 mutation database^40^: Q53X, I80T, I84S, F167X, and W204X, found in type I BPES patients showing both POI and eyelid malformation, and N105S and N109K, found in type II BPES patients exhibiting eyelid malformation, but not POI (Fig. 7a). We examined NHEJ and HR activities of these pathogenic mutants. The BPES type I mutants showed defective NHEJ activity compared to WT FOXL2; we observed complete loss of NHEJ inhibition for Q53X, I80T, and I84S, and partial loss of NHEJ inhibition for F167X and W204X (Fig. 7b and Supplementary Fig. 16a). In contrast, the BPES type II mutants (N105S and N109K) did not significantly compromise NHEJ repair (Fig. 7b and Supplementary Fig. 16a). Opposite trends were observed for HR repair activity of the BPES type I mutants; complete loss of HR stimulation was observed for Q53X, I80T, and I84S, and partial loss of HR stimulation for F167X and W204X (Fig. 7c and Supplementary Fig. 16b). Consistent with their effects on NHEJ, the type II BPES mutants N105S and N109K had not significant influence on HR efficiency (Fig. 7c and Supplementary Fig. 16b).

Next, we conducted an IP assay using these FOXL2 mutants and XRCC5/6 to investigate whether the loss of inhibitory action on NHEJ activities in the BPES type I mutants involves a defective interaction with XRCC5/6. As shown in Fig. 7d, mutants that had completely (Q53X, I80T, and I84S) or partially (F167X and W204F) lost NHEJ-inhibitory activity exhibited no or weaker interaction with both XRCC5 and XRCC6, respectively, whereas the N105S and N109K mutants had similar affinity for XRCC5/6 as the WT.

In addition, the C134W mutant of FOXL2, which is found in virtually all ACGT patients, was examined for its influence on NHEJ and HR. The C134W mutant exhibited less effective NHEJ inhibition and stronger HR stimulation (Fig. 7e, f and Supplementary Fig. 16c, d). The absolute amounts of C134W-bound XRCC5/6 proteins were clearly lower than those of WT-bound proteins, as indicated by immunoblot assay (Fig. 7g; blots on the left). However, after normalisation to the amount of immunoprecipitated C134W, the ratios of XRCC5/6 proteins bound with C134W mutant were similar to those bound to the WT (Fig. 7g; graph on the right). This phenomenon is attributable to preferential proteasome-mediated degradation of the C134W mutant through hyperphosphorylation and ubiquitination, as reported previously^25^. None of these disease-associated FOXL2 mutants induced DNA damage per se (Supplementary Fig. 16e, f).

Moreover, we analysed changes in the level of lysine-acetylation of pathogenic FOXL2 mutants. Compare to the levels of acetylated WT, the mutant FOXL2s, I80T and W204X, which are found in the type I BPES patients, showed markedly decreased acetylation, and similar levels of acetylation were found in the N109K FOXL2 mutant, which is associated with the type II BPES (Supplementary Fig. 17a). In addition, AGCT-associated C134W mutant showed decreased acetylation compared to WT (Supplementary Fig. 17b). Thus, these results further corroborate our findings on changes in the NHEJ activity among the type I and II of BPES and AGCT.

### FOXL2 competes for the same binding on XRCC5 and XRCC6

We next investigated how FOXL2 forms a mutually exclusive heterodimeric complex with XRCC5/6. We tried crystallisation of the FOXL2-XRCC6 complex, but failed to produce crystals. Alternatively, we adopted protein-protein docking approach.The starting model of XRCC6 was obtained from full-length XRCC6 (PDB ID: 1JEY)^41^ and that of FOXL2 FH domain (K124Q) was homology-modelled by using human FOXN1 model (PDB ID: 5OCN). To increase accuracy of protein-protein docking, cross-linking mass spectrometry (XL-MS) was used to gather data for intermolecular distance restraints^42^ (Supplementary Fig. 18a-c). Consistent with the IP data (Fig. 1g), two of the crosslinked residues are located on the Ku core domain of XRCC6, indicating that indeed the Ku core domain contributes to bind FOXL2 (Supplementary Fig. 18d, e). Consequently, a reliable docking model with low energy score (−132 kcal/mol) was produced (Fig. 7h).

When we examined the docking model of the FOXL2-XRCC6 complex, the protruding loop of FOXL2 containing Gln124 is reasonable docked to inner cleft of the Ku core domain and C-terminus is surface-exposed so that it can be further connected to the remaining part of FOXL2 (Fig. 7h). Importantly, FOXL2 was attached to a XRCC5-occupied site of XRCC6 (Fig. 7h; upper model), suggesting that FOXL2 and XRCC5 competes for the same binding site on XRCC6. Indeed, when the structure of the XRCC5-XRCC6 complex was superimposed on that of the FOXL2-XRCC6 complex, FOXL2 partially overlaps with XRCC5 (Fig. 7h; lower model). Collectively, these results suggest that FOXL2 and XRCC5 or XRCC6 competitively binds to XRCC6 or XRCC5, respectively, to regulate the formation of stable XRCC5–XRCC6 complex.

## Discussion

The key molecular event at DSB sites destined for either the NHEJ or the HR pathway is the recruitment of the heterodimeric Ku complex comprised of XRCC5 and XRCC6 to DSBs, which promotes NHEJ initiation and prevents additional DSB end resection that facilitates HR. However, it remains to be unknown how Ku recruitment to DSB ends is specifically regulated. Considering that XRCC5 and XRCC6 are very abundant in cells and their Ku heterodimer has a remarkably high binding affinity for DNA ends, a well-refined regulatory system that allows tight control of Ku complex recruitment to DSB regions for NHEJ must exist to avoid their random association with DSBs. The present study revealed the existence of such a regulatory circuit directed by SIRT1-mediated post-translational modification of FOXL2, which controls the Ku complex formation between XRCC5 and XRCC6 and Ku recruitment to DSB sites, allowing refined control of the DSB repair process (Fig. 7i). Upon the introduction of DSBs, the deacetylase SIRT1 is translocated to the nucleus where it binds and deacetylates the K124 residue of FOXL2, leading to the release of XRCC5/6 from FOXL2 (Fig. 7i). The freed XRCC5/6 proteins form the Ku complex, which is readily recruited to DSB sites to commence NHEJ repair (Fig. 7i).

SIRT1, a highly conserved nicotinamide adenine dinucleotide-dependent protein deacetylase, regulates diverse cellular processes, including DNA damage, genome stability, organ development, senescence, stress responses, and inflammation^43,44^. SIRT1 associates with FOXL2 and can suppress apoptotic and transcriptional activities of FOXL2 on its target genes associated with DNA repair, cell cycle, and oxidative stress response^45,46^. However, it remained unknown whether the deacetylase activity of SIRT1 toward FOXL2 directly regulates its role in maintaining genome integrity or global transcription. Here, we found that SIRT1 translocates into the nucleus in response to DNA damage, which enables increased interaction with nuclear FOXL2, which in turn allows for efficient deacetylation of FOXL2 at K124, leading to NHEJ-mediated DSB repair induction. Although further studies are needed to elucidate how DNA damage signals the nuclear translocation of SIRT1, our data uncovered SIRT1 as a key regulator of NHEJ repair and genome maintenance via the SIRT1-FOXL2-XRCC5/6 axis.

Improper DSB repair can lead to the accumulation of mutations, chromosome rearrangements, or even carcinogenesis in mammals. DSB repair pathway choice is critical for maintaining DSB repair kinetics and genome stability by controlling the balance between NHEJ and HR. Although not well understood, DSB repair pathway choice appears to be regulated by proteins that are exclusively expressed in NHEJ or HR, undergo post-translational modification in various cell-cycle phases, and differentially interact with partners^47^. We found that in addition to the direct regulatory role of FOXL2 in NHEJ, FOXL2 controls HR-mediated DSB repair activity (Fig. 3h, i) possibly through indirect regulation of the molecular recognition process directing Ku complex formation and its recruitment to DSB sites. Abundant FOXL2 protein efficiently suppresses NHEJ and enhances HR activities, whereas lack of or defective FOXL2 promotes NHEJ repair, but inactivates HR, suggesting that non-functional FOXL2 or low abundance of FOXL2 directs DSB repair to NHEJ rather than HR. The HR-stimulatory action of FOXL2 is likely an indirect effect; deacetylated FOXL2-driven release of XRCC5 and XRCC6 allows Ku association to DSB sites, which would hinder DNA-end resection, which in turn allows loading of the recombination machinery, and thus, decreases HR efficiency. This notion is further supported by the increase in chromatid-type aberrations in FOXL2-depleted cells (Fig. 6c and Supplementary Fig. 15b), because impaired HR-mediated DSB repair primarily aggravates chromatid-type aberrations^48,49^. Although direct regulation of HR by FOXL2 cannot be excluded, the present study unravelled FOXL2 as a key modulator of the balance between NHEJ- and HR-mediated DSB repair pathways.

The pivotal role of FOXL2 in ovary development is clearly demonstrated by POI development in both BPES type I patients bearing heterozygous *FOXL2* mutations^20^ and *FOXL2* heterozygous knockout mice^14,19^ as well as by the C134W FOXL2 mutation found in virtually all AGCT patients^21^. However, the pathophysiological mechanisms of POI and AGCT are not clearly understood. By defining a central function of FOXL2 in guarding genome stability by directing DSB repair pathway choice, the present study unveiled another layer of pathogenesis, involving genome instability in ovarian cells as a consequence of loss of or insufficient function of NHEJ and HR repair pathways by the ovarian disease-associated FOXL2 mutations. Infertility-inducing BPES type I FOXL2 mutants (Q53X, I80T, I84S, F167X, and W204F) show defective NHEJ and HR regulation mainly due to the decreased acetylation of FOXL2 leading to defective XRCC5/6-sequestering activities (Fig. 7b–d and Supplementary Fig. 16a, b). The AGCT-associated FOXL2 mutant C134W exhibits insufficient XRCC5/6 sequestering because of its lower steady-state level, leading to an imbalance between NHEJ and HR (Fig. 7e–g). Thus, we reason that these FOXL2 mutants fail to carry out their distinct role in switching the DSB repair mode from NHEJ to HR via Ku complex regulation.

The current study provided direct evidence of the existence of an exquisite molecular regulatory machinery that controls the balance between NHEJ and HR by a post-translational modification of FOXL2. Further extended investigation of the influence of other paralogs of FOXL2 on DBS repair activities will highlight this regulation of DBS repair choice as a central function for FOX family transcriptional factors in general.

## Methods

### Plasmids

pET28a-FOXL2, p3XFLAG-CMV-10-XRCC5, pcDNA6-HA-XRCC6 WT, and various XRCC6 mutant plasmids were generated^50,51^. Plasmids carrying mutant XRCC5 were produced by PCR using the following primers (Cosmo Genetech, Seoul, Korea): XRCC5 N-F (5′-GCAGCAGAATTCAATGGTGCGGTCGGGG-3′) and XRCC5 N-R (5′-GCAGCAGGATCCCTAAATTTTCTTGAAG-3′); XRCC5 Ku-F (5′-GCAGCAGAATTCAATGGAGAGGCATTCC-3′) and XRCC5 Ku-R (5′-GCAGCAGGATCCCTAAGCAGTCACTTG-3′); XRCC5 C-F (5′-GCAGCAGAATTCAATGCAGGAAATTTTC-3′) and XRCC5 C-R (5′-GCAGCAGGATCCCTATATCATGTCC-3′). PCR products were digested with *Xho*I and *Eco*RI (Takara Bio, Shiga, Japan) and were ligated into p3XFLAG-CMV-10 (Sigma-Aldrich, St. Louis, MO, USA). Plasmids carrying Myc-tagged FOXL2 WT, C134W, ΔAla, ΔFH, FH, K36R, K48R, Q53X, I80T, I84S, N105S, and N109K were prepared^17,18,51^. Myc-tagged K124R, K150R, K246R, K124Q, F167X, and W204X expression plasmids were produced by recombinant PCR using the following primers: FOXL2-F (5′-CTAGAATTCAAATGATGGCCAGCTACCCC-3′), K124R-F (5′-GAGCGCCGCGGCAACTACTGGACG-3′), K124R-R (5′-GTAGTTGCCGCGGCGCTCGCCGCC-3′), K150R-F (5′-CGCATGCGCAGGCCCTTCCGGC-3′), K150R-R (5′-AAGGGCCTGCGCATGCGGCGGCG-3′), K246R-F (5′-GTGGTCCGCGGGCTGGCGGGCC-3′), K246R-R (5′-GCCAGCCCGCGGACCACAGCGGC-3′), K124Q-F (5′-GAGCGCCAGGGCAACTACTGGACG-3′), K124Q-R (5′-CCAGTAGTTGCCCTGGCGCTCGCC-3′), F167X-R (5′-CTACTCGAGTTAGAGCCCCTTGCCGGGCTGGAA-3′), W204X-R (5′-CTACTCGAGCTACGAGTTGTTGAGGAAGCCAGA-3′), and FOXL2-R (5′-CTACTCGAGTCAGAGATCGAGGCGCGAATG-3′). The PCR products were digested with *Eco*RI and *Xho*I and were ligated into pCMV-Myc (Clontech, Palo Alto, CA, USA). pET30a-XRCC5 and -XRCC6 were produced by PCR using the following primers: XRCC5-F (5′-GGAATTCCATATGGTGCGGTCGGGGAAT-3′) with XRCC5-R (5′-CCGCTCGAGTATCATGTCCAATAAATC-3′) and XRCC6-F (5′-GGAATTCCATATGTCAGGGTGGGAGTCA-3′) with XRCC6-R (5′-CCGCTCGAGGTCCTGGAAGTGCTTGGT-3′). pBabe-puro-SFP-SIRT1-WT and pBabe-puro-SFP-SIRT1-H363Y were generous gifts from Dr. Ja-Eun Kim (Kyung Hee University, Seoul, Korea). SIRT1-WT and -H363Y were subcloned into p3XFLAG-CMV-10 after PCR with the following primers: SRIT1-F (5′-ATTTGCGGCCGCTTTAATGGCGGACGAGGCGGC-3′) with SRIT1-R (5′-CGCGGATCCGCGCTATGATTTGTTTGATGG-3′).

For recombinant protein expression of the FH domain of FOXL2 and its mutated form, constructs comprising residues 46-158 were cloned into pGST2 vector, using the following primers: FOXL2-FH-F (5′-GCTATATGAATTCAACCGGAGAAGCCGGACCCG-3′) and FOXL2-FH-R (5′-GCTAATTCTCGAGTCACGCGGGCGGCGGCCGG-3′). The PCR products were digested with *Eco*RI and *Xho*I and were ligated into pGST2 vector. The K124Q and K124R mutants were produced by site-directed mutagenesis using the following primers: FH-K124Q-F (5′-GCGAGGGCGGCGGCGAGCGCCAGGGCAACTACTGGACGCTGGA-3′, FH-K124Q-R 5′-TCCAGCGTCCAGTAGTTGCCCTGGCGCTCGCCGCCGCCCTCGC-3′), FH-K124R-F (5′-GCGAGGGCGGCGGCGAGCGCCGTGGCAACTACTGGACGCTGGA-3′), and FH-K124R-R (5′-TCCAGCGTCCAGTAGTTGCCACGGCGCTCGCCGCCGCCCTCGC-3′).

### Cell culture and transfection

Human adult-type granulosa cell tumour-derived KGN cells (Riken, Tsukuba, Japan), 293T human embryonic kidney cells (ATCC, Manassas, VA, USA), human juvenile granulosa cell tumour-derived COV434 cells (Sigma-Aldrich), HeLa human cervical cancer cells (Korean Cell Line Bank, Seoul, Korea), and A549 human lung adenocarcinoma cells (ATCC) were cultured in Dulbecco’s modified Eagle’s medium (DMEM) (Caisson, North Logan, UT, USA) and DMEM/F12 (Caisson) containing 10% foetal bovine serum (FBS) and 1% penicillin-streptomycin (Caisson) at 37 °C in 5% CO_2_. MRC-5 human lung normal cells (Korean Cell Line Bank) were cultured in Minimum Essential Medium containing 25 mM HEPES, 25 mM NaHCO3, 10% FBS, and 1% penicillin-streptomycin. The human B-lymphocyte cells line (GM01029; Coriell Institute, Camden, NJ, USA) were cultured in RPMI 1640 containing 15% FBS and 1% penicillin-streptomycin. Cells were transfected with Lipofectamine 2000 or 3000 (Invitrogen) according to the manufacturer’s instructions.

### RNA interference

The siRNA sequences against FOXL2 (5′-GCUCCUGUCGCUCCUCUUU-3′), SIRT1 (5′-ACUUUGCUGUAACCCUGUA-3′), and scrambled siRNAs (SN-1001-CFG) were purchased from Bioneer (Daejeon, Korea). Cells were transfected with siRNAs using Lipofectamine 2000 or 3000 (Invitrogen) according to the manufacturer’s instructions^17^.

### Reagents and antibodies

Etoposide, MMS, TSA, NIM, and thymidine were purchased from Sigma-Aldrich. Hydrogen peroxide (H_2_O_2_) was purchased from Duksan Pure Chemicals (Ansan, Korea). The following antibodies were used in this study: anti-XRCC5 (sc-5280; Santa Cruz Biotechnology, Santa Cruz, CA, USA; 1:1000), anti-XRCC6 (sc-55505; Santa Cruz Biotechnology; 1:1000), anti-β-actin (sc-47778; Santa Cruz Biotechnology; 1:3000), anti-γ-H2AX (#9718S; Cell Signaling Technology, Danvers, MA, USA; 1:1000), anti-PARP-1 (sc-74469; Santa Cruz Biotechnology; 1:1000), anti-α-tubulin (LF-PA0146; AbFrontier, Seoul, Korea; 1:1000), anti-SIRT1 (sc-74504; Santa Cruz Biotechnology; 1:1000), anti-Myc (#2276 S; Cell Signaling Technology; 1:1000), anti-Flag (#2368S; Cell Signaling Technology; 1:1000), anti-HA (sc-805; Santa Cruz Biotechnology; 1:1000), anti-His (sc-803; Santa Cruz Biotechnology; 1:1000), anti-acetylated lysine (sc-32268; Santa Cruz Biotechnology; 1:1000), and anti-phosphorylated serine (sc-81514; Santa Cruz Biotechnology; 1:1000). Polyclonal anti-FOXL2 antibodies (1:1000) were generated using either recombinant human FOXL2 protein or N-terminus peptides as an antigen^51^. Other reagents were purchased from Sigma-Aldrich, unless otherwise indicated. Antibodies were validated for the specific species and application in pilot studies.

### Generation of the FOXL2 antibody

Rosetta 2 (DE3) (EMD Millipore, Billerica, MA, USA) competent cells, a derivative strain of *Escherichia coli* strain BL21, containing pET28a-FOXL2 was cultured to an OD_600_ of 0.4–0.6 and 0.1 mM IPTG was added to the cultures. After incubation, the cells were lysed and the 6×His-tagged human recombinant FOXL2 protein was purified by affinity chromatography with Ni-NTA agarose beads (Qiagen, Valencia, CA, USA) and eluted using an imidazole gradient (5–400 mM)^25,51^. One female rabbit (New Zealand white, 6-weeks, 2 kg) received a primary immunisation of 500 μl immunogen (1 mg of purified FOXL2 protein dissolved in PBS) mixed with an equal volume of Freund’s complete adjuvant (FCA) (Sigma) by subcutaneous injection. For subsequent immunisations, the rabbit was immunised with 500 μl of FOXL2 immunogen mixed with an equal volume of Freund’s incomplete adjuvant (FIA) every 2 week. After 4 immunisations, the rabbit serum was harvested and purified by Protein A Agarose (Thermo Fisher Scientific, Waltham, MA, USA) followed by affinity purification with purified recombinant FOXL2 protein using AminoLink Immobilization Kit (Thermo Fisher Scientific).

### TAP and LC-MS/MS

The 293T cells (1 × 10^8^) were transfected with 30 μg of a pNTAP plasmid encoding streptavidin-binding peptide (Sbp) and calmodulin-binding peptide (Cbp) with or without FOXL2. After 24 h of incubation, the cells were used for the fractionation of cytosolic and nuclear proteins^25^. Each 200 μg of the nuclear proteins from the FOXL2-NTAP plasmid- and empty vector-containing cells were applied for streptavidin- and calmodulin-based TAP as described elsewhere^52^. Affinity purified nuclear proteins were visualised by SDS PAGE and silver staining, after which FOXL2 and interacting proteins from the FOXL2-NTAP plasmid-containing cells were identified compared to those from the empty vector-containing control cells. Using the different protein bands that were excised from a gel containing FOXL2 and interacting proteins purified by TAP, tryptic peptides were prepared for the identification of proteins by tandem mass spectrometry as described elsewhere^53^. Tryptic peptides were analysed on the Thermo Scientific LTQ XL linear ion trap mass spectrometer connected to a reversed-phase Magic C18AQ column (ID75 × OD150 μm × L75 mm) in the Agilent Series 1200 system operated at a flow rate of 0.4 μl min^−1^. The chromatographic condition was a 55-min linear gradient from 5 to 40% acetonitrile in 0.1% formic acid, and followed by a 10-min column wash with an 80% acetonitrile concentration and a 20-min re-equilibration to the initial buffer condition. A blank run was made prior to each sample injection. Survey full-scan mass spectrometry spectra (*m/z* 300–2000) were performed to determine the MS2 precursor ions and charge states. The MS2 spectra of the six most intense ions from the preview survey scan were acquired in the ion trap with the following options: isolation width 1.5 Da; collision energy 25%; and dynamic exclusion duration 30 s. The acquired mass spectral data were manipulated with Xcalibur software and identified with expectation log(e) values less than −2 from a non-redundant sequence database of *Homo sapiens* (women) using X!Tandem at http://www.thegpm.org/. The following search options were used: precursor mass tolerance, 4 Da; fragment mass error, 0.4 Da; fixed carbamidomethylation of cysteine (C); and refined searches for oxidation of methionine (M) and tryptophan (W), phosphorylation of serine (S), threonine (T) and tyrosine (Y), deamidation of asparagine (N) and glutamine (Q), and *N*-acetylation of lysine (K).

### Immunoprecipitation (IP) and immunoblot analysis

Cells transfected with the indicated plasmids or treated with the indicated reagents were lysed, and IP and immunoblotting were conducted^54^. In brief, the cell lysates were prepared and immunoprecipitated with indicated antibodies (1 μg) coupled with 20 μl of Dynabeads protein G (Invitrogen) according to manufacturer’s instructions. After incubation, the samples were boiled and subjected to SDS-PAGE for immunoblotting with the respective antibodies. The membranes were detected using an Amersham Imager 600 (GE Healthcare Life Sciences, Amersham, Buckinghamshire, UK) and the intensity of each band was quantified using Quantity One software (Bio-Rad Laboratories, Hercules, CA, USA). All of the uncropped blots are provided in the Source Data file.

### Immunocytochemistry

KGN cells were seeded onto 12-mm cover slips, and DSBs were induced after 24 h of culture. The cells were fixed with 4% paraformaldehyde (Sigma-Aldrich) and penetrated with 0.2% Triton X-100 (Sigma-Aldrich) in PBS with 0.1% Tween-20 (PBST). The cells were blocked with 1% bovine serum albumin (BSA) in PBST for 1 h at room temperature. After washing with PBST three times, the cells were immunostained with the specific antibodies (anti-FOXL2 [1:50], anti-XRCC5 [1:200], anti-XRCC6 [1:100], or anti-SIRT1 [1:100]) in PBST containing 2.5% BSA for overnight at 4 °C. The cells were stained with rabbit IgG labelled with Alexa 488 (Invitrogen) or mouse IgG labelled with Alexa 546 (Invitrogen). Fluorescence was detected using a laser scanning confocal microscope (LSM 800; Carl Zeiss, Gottingen, Germany). Colocalisation of two proteins was quantified using a Zen software (Carl Zeiss).

### Recombinant protein purification

Rosetta 2 (DE3) competent cells (EMD Millipore) transformed with pET30a-XRCC5 or -XRCC6 were cultured to an OD_600_ of 0.5. Then, 0.1 mM isopropyl β-d-thiogalactoside (IPTG) was added to the cultures. After 3 h, the cells were harvested and resuspended in lysis buffer (50 mM NaH_2_PO_4_, 300 mM NaCl, and 5 mM imidazole, pH 8.0), and lysed by sonication. The His_6_-tagged XRCC5 and XRCC6 proteins were purified by affinity chromatography with Ni-NTA agarose beads (Qiagen) and eluted using an imidazole gradient (5–400 mM).

For BLI and cross-linking mass spectrometry experiments, constructs of FOXL2-FH domain (K124, K124Q, and K124R) comprising residues 46-158 were cloned into pGST2 vector, which contains a N-terminal GST tag. The proteins were expressed in *E. coli* BL21 (DE3) cells induced with 0.5 mM IPTG. For cell lysis, cell pellets were resuspended in buffer A (20 mM Tris-HCl pH 8.0 and 200 mM NaCl) containing 1 mM phenylmethylsulfonyl fluoride. Cells were lysed with a microfluidizer (Microfluidics, Westwood, MA, USA) and then lysed cells were centrifuged at 4600 × *g* (Vision V506CA rotor) for 30 min at 277 K to pellet the cell debris; the supernatant was applied to a glutathione-Sepharose column (GE Healthcare, Little Chalfont, UK) pre-equilibrated with buffer A. Proteins were eluted with buffer A containing 15 mM reduced glutathione. The eluted protein was loaded directly onto a 5 ml Hi-Trap heparin (GE) column that had been equilibrated in buffer A, and eluted using a linear gradient of 0.2–1 M NaCl in buffer A. The eluates were cleaved by TEV protease, and loaded through a 5 ml Hi-Trap SP (GE) column pre-equilibrated with buffer A to remove the GST tag, followed by gel filtration on a HiLoad 16/60 Superdex 75 column (GE). For His_6_-tagged XRCC6, which was cloned into pET30a, proteins were expressed in *E. coli* Rosetta2 pLysS competent cells (EMD). The protein was purified using a Ni-affinity column (GE), followed by 5 ml Hi-Trap heparin (GE) column that had been equilibrated in 20 mM Tris-HCl pH 8.0 and 100 mM NaCl. The protein was eluted using a salt gradient (0.1–1 M NaCl).

### Size-exclusion chromatography with multi-angle light scattering

Size-exclusion chromatography with multi-angle light scattering (SEC-MALS) experiments for XRCC6 (aa 1-609) and the FH domain of FOXL2 (aa 46-158) were performed using FPLC system (GE Healthcare) connected to a Wyatt MiniDAWN TREOS MALS instrument and a Wyatt Optilab rEX differential refractometer (Wyatt Technology, Santa Barbara, CA, USA). A Superdex 200 10/300 GL (GE Healthcare) gel filtration column pre-equilibrated with buffer A, was normalised using ovalbumin. Proteins were injected at a flow rate of 0.4 ml/min. Data were analysed using the Zimm model for static light-scattering data fitting, and graphs were constructed using EASI graph with a UV peak in the ASTRA V software (Wyatt Technology).

### NHEJ DNA repair assay

293TdA3 cells containing chromosomally integrated pIRES-TK-EGFP^32^ were transfected with the indicated plasmids or siRNAs. After 24 h, pCBA*Sce*I^32^, an I-*Sce*I endonuclease expression vector, was introduced into the 293TdA3 cells. EGFP fluorescence was measured after 24 h using a FlexStation 3 microplate reader (Molecular Devices, Sunnyvale, CA, USA). In addition, genomic DNA was purified, and uncut or joined DNAs were analysed by qPCR) using primers pC (5′-CGTTTGCCCGGGAGATG-3′) and pD (5′-CGACCGGTAGGCGTTATCAG-3′), and JP-F1 (5′-CGTACGTCTCCGGATTCGAA-3′) and JP-R1 (5′-GTGATGCGGCACTCGATCTT-3′), respectively. The proportion of “uncut DNA” is the amount of DNA left to be uncleaved by I-*Sce*I, which, at the same time, reflecting the amount of DSBs generated by I-*Sce*I, while the proportion of “joined DNA” indicates the degree of ligation efficiency of the DBS ends by NHEJ repair.

### HR DNA repair assay

293T-DR-GFP cells containing chromosomally integrated pDR-GFP (Addgene plasmid # 26475; http://n2t.net/addgene:26475; RRID:Addgene_26475) were transfected with indicated plasmids or siRNAs for 24 h. Subsequently, the 293T-DR-GFP cells were transfected with the pCBA*Sce*I DNA plasmid for 24 h. GFP fluorescence was measured with the FlexStation 3. Genomic DNA was purified, and recombined DNA was analysed by PCR^55^. Unrecombinant and recombinant primer sets for PCR amplifications used were: unrec (5′-GCTAGGGATAACAGGGTAAT-3′) with reverse primers (5′-TGCACGCTGCCGTCCTCG-3′), and rec (5′-GAGGGCGAGGGCGATGCC-3′) with the reverse primers, respectively.

### ChIP assay

293TdA3 cells were transfected with indicated plasmids or siRNAs for 12 h. Then, pCBASceI was introduced into the 293TdA3 cells. After 24 h of incubation, cells were crosslinked with 1% formaldehyde and lysed with SDS buffer. The lysates were further sonicated and immunoprecipitated with indicated antibodies. The immunoprecipitated complexes were collected using protein G-agarose bead (Invitrogen) and, cross-linking was reversed by incubation at 65 °C. The proteins were digested with proteinase K, and the DNA was purified. To analyse the damaged regions of DNA, the DNA was amplified using the following primers 0.3-kb regions (forward: 5′-ATGGGCTACGGCTTCTACCA-3′ and reverse: 5′-GCCGTCCTCGTACTTCTCGAT-3′), 1.2-kb regions (forward: 5′-GGTGTGCGTTTGTCTATATGTGATT-3′; and reverse: 5′-CCTAGGAATGCTCGTCAAGAAGA-3′), and 2.5-kb regions (forward: 5′-CCCTGAACCTGAAACATAAAATGA-3′; and reverse: 5′-TGTGAAATTTGTGATGCTATTGCTT-3′)^51^.

### Generation of CRISPR/Cas9-nickase-mediated *FOXL2* KO cells

FOXL2 KO cells were generated following the protocol of Ran et al^34^. Briefly, to generate a Cas9 (D10A) nickase-encoding vector, pSpCas9n(BB)-2A-GFP was mutagenized by PCR using pSpCas9(BB)-2A-GFP (Addgene plasmid #48138; http://n2t.net/addgene:48138; RRID:Addgene_48138) as a template (Addgene, Cambridge, MA, USA). The primers used for site-specific mutagenesis were D10A-F (5′-TAGAGGTACCCGTTACATAAC-3′), D10A-R (5′-CTGAAGATCTCTTGCAGATAG-3′), Mut-D10A-F (5′-TACAGCATCGGCCTGGCCATCGGC-3′), and Mut-D10A-R (5′-GGTGCCGATGGCCAGGCCGATGCT-3′). Paired single-guide RNA-1 and RNA-2 that target the forkhead region of FOXL2 were designed using CIRSPR DESIGN (http://crispr.mit.edu/). To generate plasmids targeting FOXL2, sense and antisense gRNA-1 (F: 5′-CACCGTGAGCGCCACGTACGAGTAC-3′, R: 5′-AAACGTACTCGTACGTGGCGCTCAC-3′) and gRNA-2 (F: 5′-CACCGGAGAAGAGGCTCACGCTGTC-3′, R: 5′-AAACGACAGCGTGAGCCTCTTCTCC-3′) oligonucleotides (Cosmo Genetech) were annealed. The products were digested with *Bbs*I (Thermo Fisher Scientific) and ligated into pSpCas9n(BB)-2A-GFP. KGN cells (1 × 10^6^) were seeded in a 100-mm dish, incubated for 24 h, and transfected with 5 µg of pSpCas9n(BB)-2A-GFP-FOXL2 forkhead-guide-1 and -2. After 24 h of incubation, the cells were suspended in basic sorting buffer (1 mM EDTA and 25 mM HEPES [pH 7.0], heat-inactivated 1% FBS, and Ca^2+^/Mg^2+^-free phosphate-buffered saline [PBS]) and sorted by flow cytometry (BD FACSAria II cell sorter; BD Biosciences, San Jose, CA, USA) based on GFP signal. The sorted cells were individually seeded into each well of a 96-well plate and further cultured for selection FOXL2 KO cell line establishment was confirmed by both western blot and genotype analyses. Allelic changes in the FOXL2 KO cell line were confirmed by TA cloning (TOPcloner™ TA core Kit; Enzynomics, Daejeon, Korea) followed by DNA sequencing analysis (Cosmo Genetech).

### BLI measurements

BLI measurements were performed to evaluate binding between XRCC6 and the FH domain of FOXL2 proteins comprising residues 46–158 (K124 and K124R) using a BLItz system (ForteBio, Menlo Park, CA, USA). C-terminal His-tagged XRCC6 (aa 1-609) was immobilised on a Ni-NTA biosensor chip surface in buffer A, and then the surface was equilibrated with buffer A. To determine if interactions occur between the FOXL2 proteins and XRCC6, the prepared Ni-NTA biosensor chip was dipped in 3–200 μM protein solution in running buffer. Both associations and dissociations were measured for 200 s. Data were collected at 100 s after the association of analyte started and dissociation constant (*K*_D_) calculation was done using GraphPad Prism program (GraphPad, San Diego, CA, USA).

### Cell cycle synchronisation

After overnight incubation of KGN cells at 30-40% confluence, the cells were treated with 2 mM of thymidine for 18 h following washing cells with PBS, and fresh medium was added. Following 9 h of incubation of the cells, 2 mM thymidine was added and the cells were cultured for additional 18 h to synchronise cells at G1/S boundary. Cells were washed with PBS, replaced with fresh media following collection at 0, 4, 9, and 14 h for cell cycle analysis by DNA staining with PI using flow cytometry (BD Biosciences).

### Ku and γ-H2AX foci analyses

Cells were seeded on 12-mm coverslips, incubated for 24 h, and irradiated with UV (CL-1000 Ultraviolet Crosslinker; UVP Inc., Upland, CA, USA). After 2 h, the cells were washed with PBS and incubated with CSK buffer containing RNase A (0.1 mg/ml) and 0.7% Triton X-100 at room temperature for 3 min^35,56^. After two washes with CSK buffer without detergent, the cells were fixed with 4% paraformaldehyde for 5 min and permeabilized with 0.2% Triton X-100 for 5 min. Then, the cells were blocked with 2.5% bovine serum albumin (BSA) in PBS with 0.1% Tween-20 (PBST) for 1 h and incubated overnight with anti-XRCC5 and anti-γ-H2AX. After three washes with PBST, the cells were incubated with Alexa 488-labelled rabbit IgG or Alexa 546-labelled mouse IgG for 1 h. Images were captured with an Eclipse Ti-E fluorescence microscope (Nikon, Tokyo, Japan) using a 100× oil objective (NA = 1.4, oil immersion, Leica). The percentage colocalisation of XRCC5 with γ-H2AX foci was quantified using NIS-elements microscope imaging software (Nikon).

### Comet assay

Cells were irradiated with UV (CL-1000 Ultraviolet Crosslinker), incubated at 37 °C, and harvested at 0 to 2 h after irradiation. A neutral comet assay was conducted^57^. In brief, cells were mixed with 1% low-gelling-temperature agarose (Sigma-Aldrich; 1:10 [v/v]). Once the agarose had solidified, the cells were lysed with lysis buffer (2% sarkosyl, 0.5 M Na_2_EDTA, and 0.5 mg/ml proteinase K, pH 8.0) at 4 °C overnight, followed by three 20-min washes with electrophoresis buffer (90 mM Tris buffer, 90 mM boric acid, and 2 mM Na_2_EDTA, pH 8.5). The proteins in the washed gels were subjected to electrophoresis at 20 V for 25 min. The slides were stained with propidium iodide (Sigma-Aldrich), and fluorescence images were captured by laser scanning confocal microscopy (Carl Zeiss). The tail moment was quantified using CASP version 1.2.3 beta2 (CaspLab, Wroclaw, Poland).

### Metaphase spread analysis

Cells were treated with 0.05 μg/ml colcemid (Sigma-Aldrich) at 21 h after UV irradiation to arrest cells in metaphase. After 3 h, the cells were harvested and treated with prewarmed hypotonic 0.075 M KCl solution at 37 °C for 10 min. Then, the cells were centrifuged and fixed with ice-cold methanol:acetic acid (3:1) solution three times. Chromosome spreads were prepared by dropping the cell suspensions on slides and were stained with Giemsa solution (BDH, London, UK). The metaphase spreads were imaged using a microscope (Leica DM750) using a 100× oil objective (NA = 1.4, oil immersion). All aberrations were scored according to published classification standards^58^.

### Cross-linking mass spectrometry

Equimolar (~100 μM) amounts of XRCC6 (aa 1-609) and the FH domain of FOXL2 (aa 46-158) were mixed in a buffer containing 20 mM HEPES pH 7.5 and 200 mM NaCl. BS3 (Thermo Fisher Scientific) was added to the protein mixture to a final concentration of 1 mM. The mixture was incubated for 30 min at room temperature with mild shaking (350 rpm) on a thermomixer (Eppendorf, Hamburg, Germany). The cross-linking reaction was quenched by adding 1 M Tris-HCl pH 7.5 to a final concentration of 50 mM. The crosslinked products were analysed by SDS-PAGE. Bands from the SDS-PAGE gels corresponding to crosslinked complexes were excised, and the proteins were reduced, alkylated, digested with trypsin (Thermo Fisher Scientific), and desalted using ZipTip C18 (Millipore, Burlington, MA, USA). Final eluates were resuspended in 0.1% (v/v) formic acid. Samples were analysed by LC-MS/MS analysis on a Q Exactive mass spectrometer (Thermo Fisher Scientific). Data analysis was performed by xQuest^59^ and Proteome Discoverer 2.3 (Thermo Fisher Scientific), using a sequence database containing the two target proteins, and validated by xProphet^60^. Crosslinks with deltaS < 0.95 were used for structural analysis.

### Molecular docking

We used HADDOCK version 2.2^61^ for protein–protein docking study. The HADDOCK was also used to refine the docked structures staring from the randomly generated initial structures. This process includes rigid body docking followed by semi-flexible searching using simulated annealing (SA), especially at the interface region. The docking process was completed by consideration of water solvation. During this process, the number of possible docked structures was narrowed down based on the docking scores. The default number of initial structures generated in rigid body docking was 1000, and 200 best structures were subject to the next semi-flexible docking. Finally, 200 possible docked structures were obtained for analysis after consideration of water solvation. For better results, we have changed this value to 2000, 400, and 200, respectively. As for the parameters for HADDOCK, we have used the default 5.4 version of protein and solvent topologies as implemented in HADDOCK 2.2 throughout the docking procedure. The starting structure of XRCC6 was obtained from Protein Data Bank (PDB ID: 1JEY) and that of FOXL2 FH domain (K124Q) was homology-modelled by using human FOXN1 model (PDB ID: 5OCN). Cross-link lengths were used to define unambiguous intermolecular distance constraints. We used active sites with no passive site for the acetylation-mimic residue of FOXL2 (Gln124) and residues (Lys357, Lys358, His360, Tyr361, Leu362, Arg363, Tyr400, Arg403, Arg404, Tyr409, Leu434, Phe436, Pro438, and Lys468) of the Ku core domain of XRCC6, based on the previous IP results. For the analysis of the docked structures, we have used fraction of common contact (FCC) based cluster analysis as incorporated in HADDOCK. In FCC, the structural similarity for clustering was based on atomic contact with pre-defined distance as contact threshold. The structure figures were plotted using the program PyMOL (http://pymol.sourceforge.net).

### Statistical analysis and reproducibility

Scatter plots and column bar graphs were generated using GraphPad PRISM (GraphPad, San Diego, CA, USA). All data are presented as the mean ± SEM (standard error of mean). Multiple comparison tests were conducted using the Student–Newman–Keuls method (SAS version 9.2; SAS Institute, Cary, NC, USA), and unpaired, two-tailed Student’s *t* test (SigmaPlot; Systat Software, San Jose, CA, USA) was used for comparisons with controls. *P* < 0.05 was considered statistically significant. All data presented with statistical analyses were obtained from three independent experiments performed triplicate except for Fig. 6. Result presented without statistical analysis were repeated as follows: Figs. 1a–d, h–j, 2a–d, 3c, 4a–e, and Supplementary Figs. 7a–c, 8a–d and 9a–c were performed as three independent experiments, Supplementary Figs. 2a–d, 6, 10 and 17 were performed as two independent experiments, and Supplementary Figs. 1a, 1b, 3a, 3b, 11, 18a and 18b were conducted once.

### Reporting summary

Further information on research design is available in the Nature Research Reporting Summary linked to this article.

## Supplementary information

Supplementary Information

Reporting Summary

## Data Availability

All data generated or analysed in this study are included in the published article and Supplementary Information file or available from the authors upon reasonable request. All raw data used for generating figures are provided as a Source Data file.

## Source data

Source Data
