# Supplementary material for: FOXL2 directs DNA double-strand break repair pathways by differentially interacting with Ku
Source: Nat Commun. 2020 Apr 24;11:2010. doi: 10.1038/s41467-020-15748-1 (PMC7181608; doi:10.1038/s41467-020-15748-1)
Supplement: Supplementary file 1 — Supplementary Information [file 41467_2020_15748_MOESM1_ESM.pdf]

Supplementary Information

FOLX2 Directs DNA Double-strand Break Repair Pathways by Differentially Interacting with Ku

Jin *et al*

Supplementary Information contains

Supplementary Tables 1 and 2

Supplementary Figures 1 – 18

Supplementary Table 1. A list of top ranked proteins determined by X!Tandem

Sample No.	Ensembl ID	NCBI RefSeq	Protein	Description	log(e)	Match % (corrected)
1	ENSP00000355759	NP_001609.2	PARP1	Poly [ADP-ribose] polymerase 1	-219.3	34
2	ENSP00000375977	NP_066964.1	XRCC5	X-ray repair cross-complementing protein 5	-13.9	4
3	ENSP00000324173	NP_005338.1	HSPA5	Endoplasmic reticulum chaperone BiP	-187.4	39
4	ENSP00000227378	NP_006588.1	HSPA8	Heat shock cognate 71 kDa protein	-594.4	67
5	ENSP00000364802	NP_005336.3	HSPA1A	Heat shock 70 kDa protein 1A	-374.9	51
6-19	ENSP00000333188	NP_075555.1	FOXL2	Forkhead box protein L2	-267.7	100

Mass spectrometry results for top rank proteins identified with more than 2 unique peptides and log(e) < -10, in which the samples no. 20 and 21 show ambiguous results of the filtering for the highest match score and lowest log(e) value.

Supplementary Table 2. List of the inter- and intra-molecular cross-links

Type	Protein 1	Position 1	Protein 2	Position 2	Cα-Cα Distance in the model (Å)
Inter	FOXL2	73	XRCC6	510	24.2
Inter	FOXL2	114	XRCC6	207	31.0
Intra	XRCC6	129	XRCC6	510	37.1
Intra	XRCC6	182	XRCC6	526	21.0
Intra	XRCC6	189	XRCC6	351	53.3
Intra	XRCC6	189	XRCC6	451	72.5
Intra	XRCC6	207	XRCC6	282	40.8
Intra	XRCC6	265	XRCC6	357	31.3
Intra	XRCC6	282	XRCC6	443	27.8
Intra	XRCC6	317	XRCC6	510	58.5
Intra	XRCC6	351	XRCC6	357	12.9
Intra	XRCC6	451	XRCC6	526	21.0
Intra	XRCC6	461	XRCC6	510	20.6

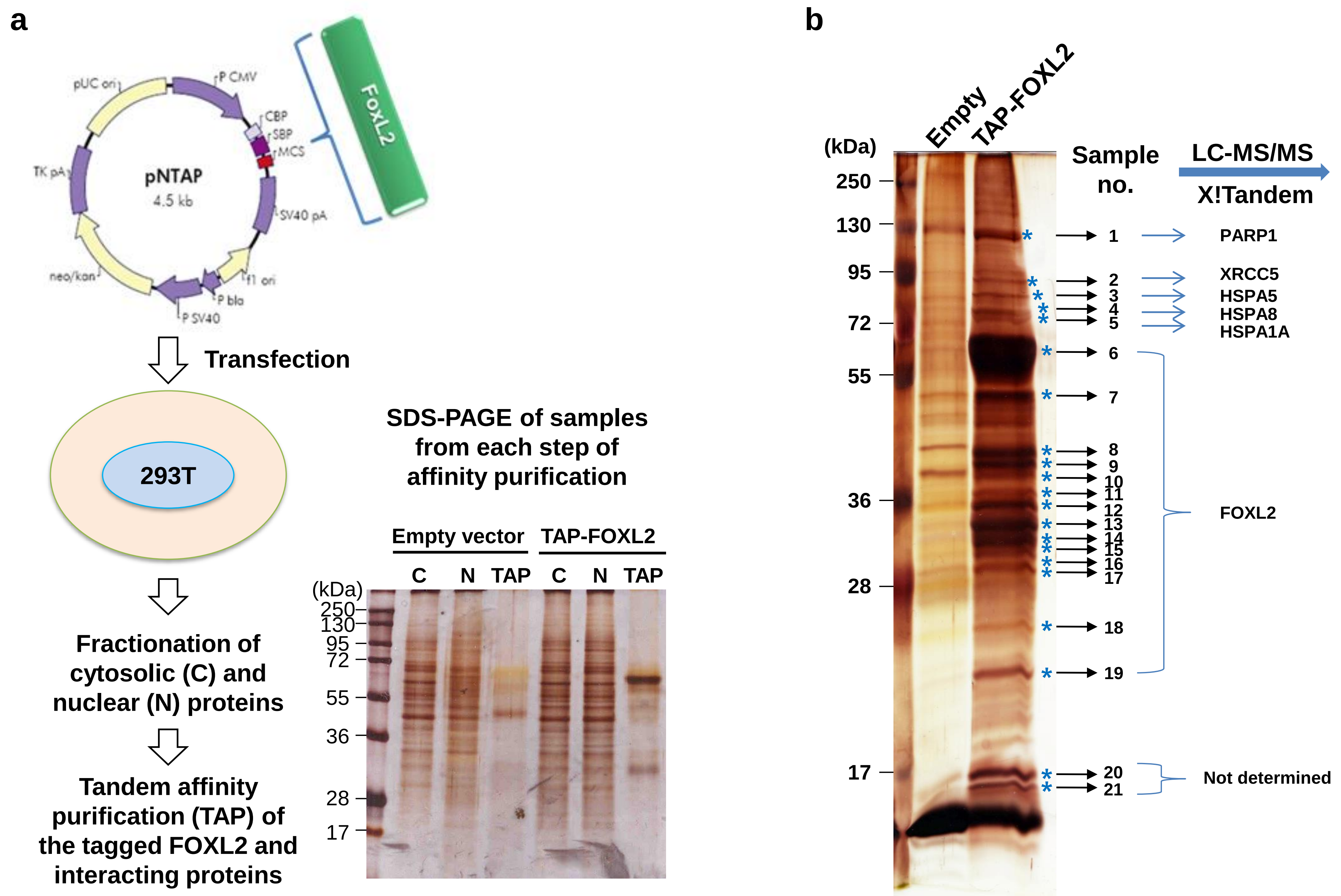

Supplementary Fig. 1. SDS PAGE analysis for the identification of FOXL2 and interacting proteins. a Tandem affinity purification of FOXL2 and interacting proteins in the nucleus of the 293T cells transfected with pNTAP plasmid encoding FOXL2 or empty vector. Equal amounts (2 μg per lane) of cytosolic (C), nuclear (N), and affinity purified (TAP) samples obtained from the TAP-FOXL2 plasmid- and empty vector-containing cells were analysed to check the quality and quantity of the samples by SDS PAGE and silver staining. **b** Identification of FOXL2 and interacting proteins in the affinity purified sample by SDS PAGE and mass spectrometry analysis. Each 200 μg amount of the nuclear proteins fractionated from the TAP-FOXL2 plasmid- and empty vector-containing 293T cells was used for tandem affinity purification. The final samples obtained from streptavidin- and calmodulin-based tandem affinity purification of the nuclear fractions of the TAP-FOXL2 plasmid- and empty vector-containing cells were compared by SDS PAGE and silver staining. Positions of the sample numbers of FOXL2 and interacting proteins identified by the gel-based mass spectrometry analysis are shown by asterisks at the right of the affinity purified sample of the TAP-FOXL2 plasmid-containing cells. Source data are provided as a Source Data file.

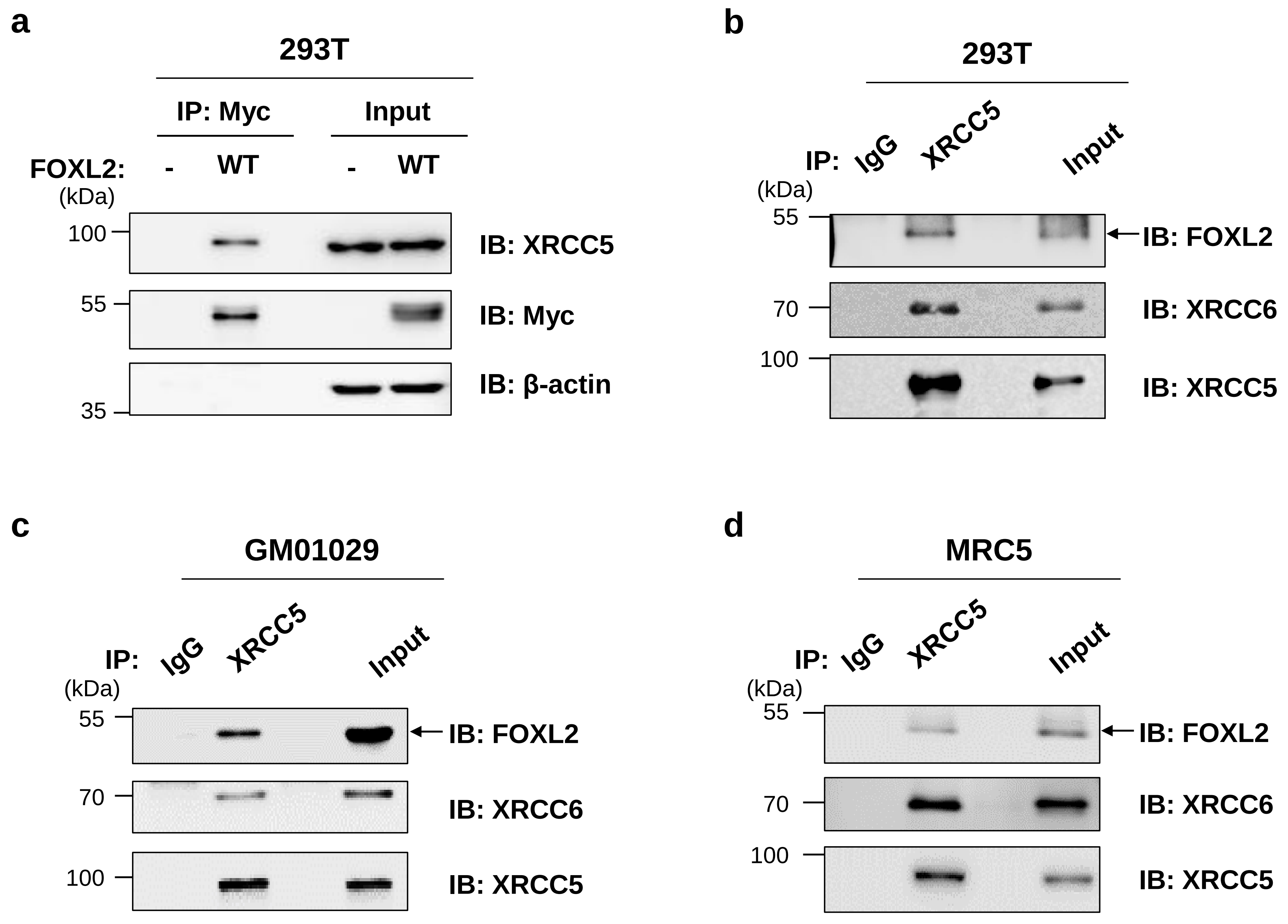

Supplementary Fig. 2. Confirmation of FOXL2 interaction with XRCC5. a 293T cells were transfected with pCMV-Myc-FOXL2 and cell lysate was immunoprecipitated with anti-Myc antibody. The immunoprecipitate was analysed by western blotting using the indicated antibodies. **b-d** Interactions between endogenously expressed FOXL2 and XRCC5/6 proteins in normal cells including 293T, GM01029, and MRC5 were demonstrated by immunoprecipitation with IgG (control) or anti-XRCC5 antibodies followed by immunoblot analysis with indicated antibodies. Source data are provided as a Source Data file.

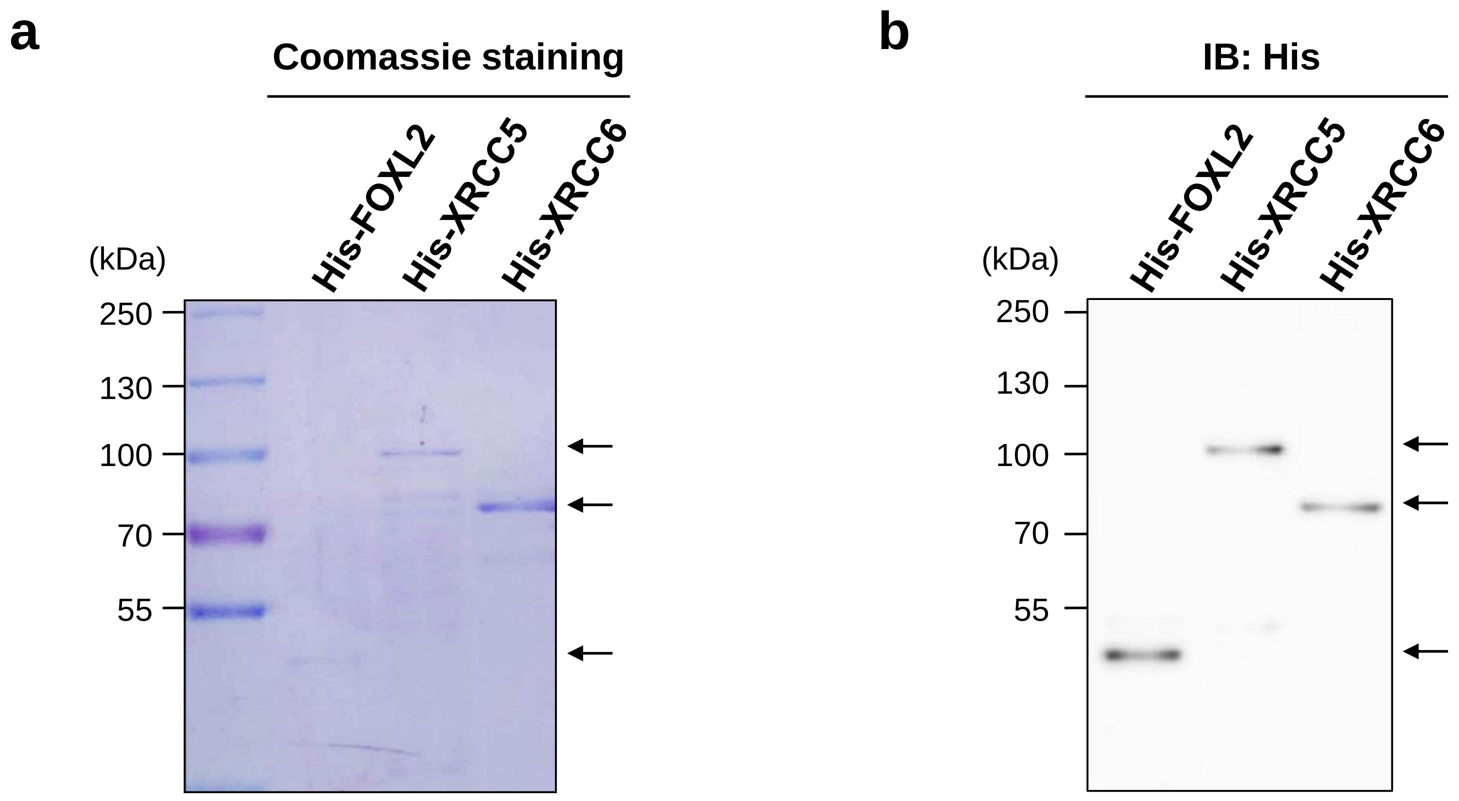

Supplementary Fig. 3. Purification of recombinant His-tagged FOXL2, XRCC5, and XRCC6. a, **b** Purified recombinant His-tagged-FOXL2, -XRCC5, and -XRCC6 were analysed by Coomassie blue staining (**a**) and immunoblot with anti-His antibody (**b**). Source data are provided as a Source Data file.

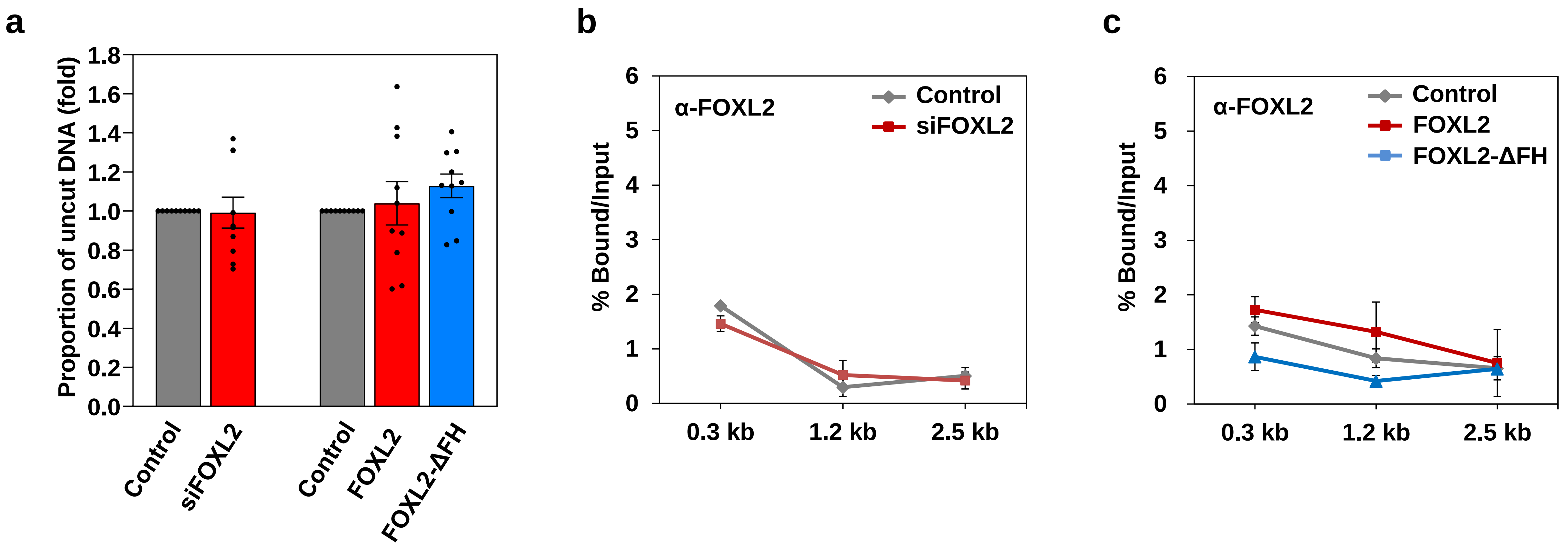

Supplementary Fig. 4. Modulations of FOXL2 expression level itself do not induce DSBs. a 293TdA3-stable cells expressing I-*Sce*I were transfected with control or FOXL2 siRNAs, or plasmid expressing empty vector (control), FOXL2, or FOXL2-ΔFH. After 48 h, the proportion of uncut DNA in the cells was measured by quantitative PCR. The proportion of "uncut DNA" is the amount of DNA left to be uncleaved by I-*Sce*I, which reflects the amount of DSBs generated by I-*Sce*I. **b**, **c** ChIP was performed with antibody against FOXL2 after transfection of control or FOXL2 siRNA (**b**), or plasmid expressing empty vector (control), FOXL2, or FOXL2-ΔFH (**c**) in I-*Sce*I expressing 293TdA3 cells. Data are the mean ± SEM of three independent experiments performed in triplicate. Source data are provided as a Source Data file.

Supplementary Fig. 5

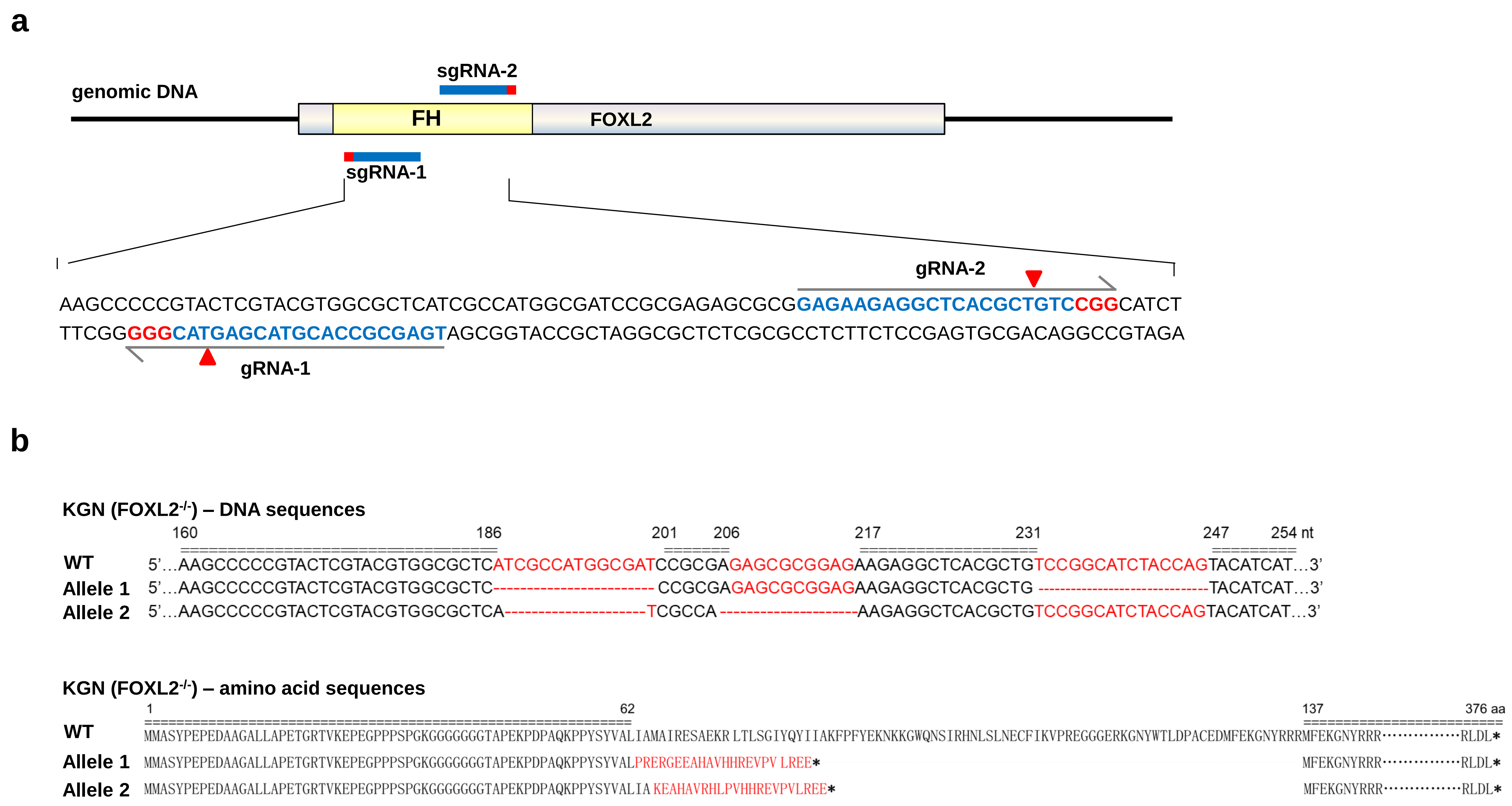

Supplementary Fig. 5. Generation of FOXL2-knockout (FOXL2$^{-/-}$) KGN cells. a Schematic representation of the strategy used for knockout of *FOXL2* in KGN cells using the CRISPR/Cas9-nickase system. A pair of guide RNAs (gRNAs) were designed to target the FH domain of FOXL2. The guide RNAs (gRNA-1 and -2) sequences (blue) and PAM sequences (red) are shown. The red triangles indicate possible cleavage sites. **b** DNA and predicted amino acid sequences around the targeting sites in the FOXL2$^{-/-}$ cell line.

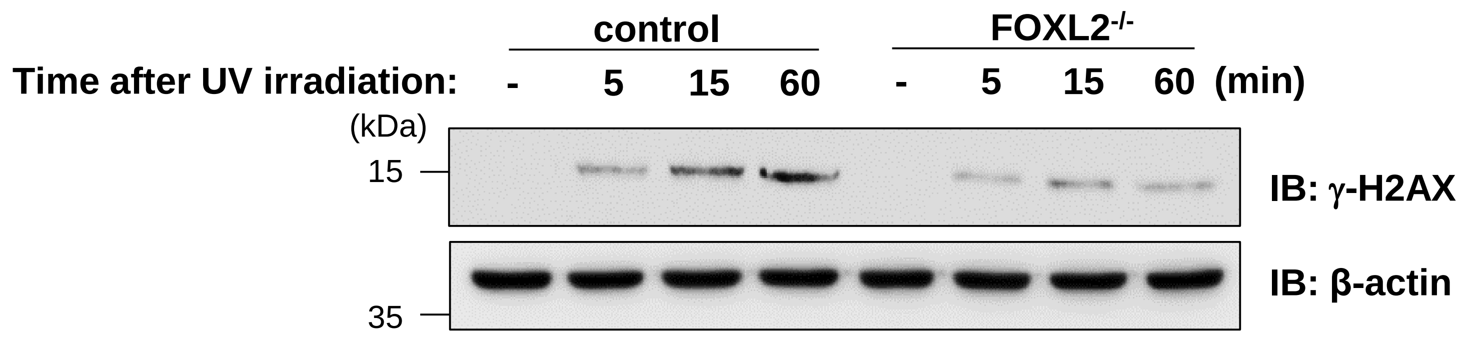

Supplementary Fig. 6. Assessment of changes in γ-H2AX level following UV irradiation. Control or FOXL2$^{-/-}$ KGN cells were irradiated with UV (100 J/m^2) and further incubated for indicated durations. Cell lysates were prepared, and western blot analysis was performed using anti- γ-H2AX antibody. Source data are provided as a Source Data file.

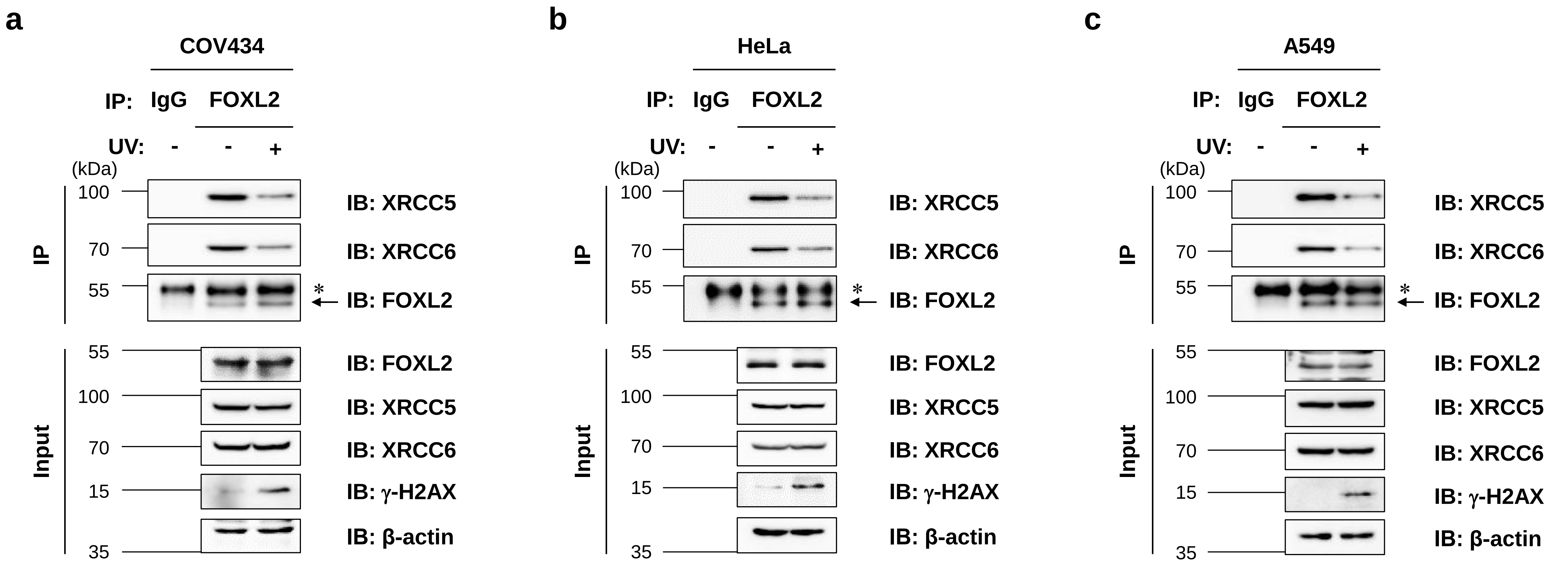

Supplementary Fig. 7. UV-induced changes in FOXL2 binding to XRCC5/XRCC6. a–c UV (100 J/m^2, 2 h)-irradiated COV434 (**a**), HeLa (**b**) and A549 (**c**) cells were subjected to immunoprecipitation with anti-FOXL2 antibody. The amounts of precipitated XRCC5 and XRCC6 were compared with those of control cells by immunoblotting using the indicated antibodies. Source data are provided as a Source Data file.

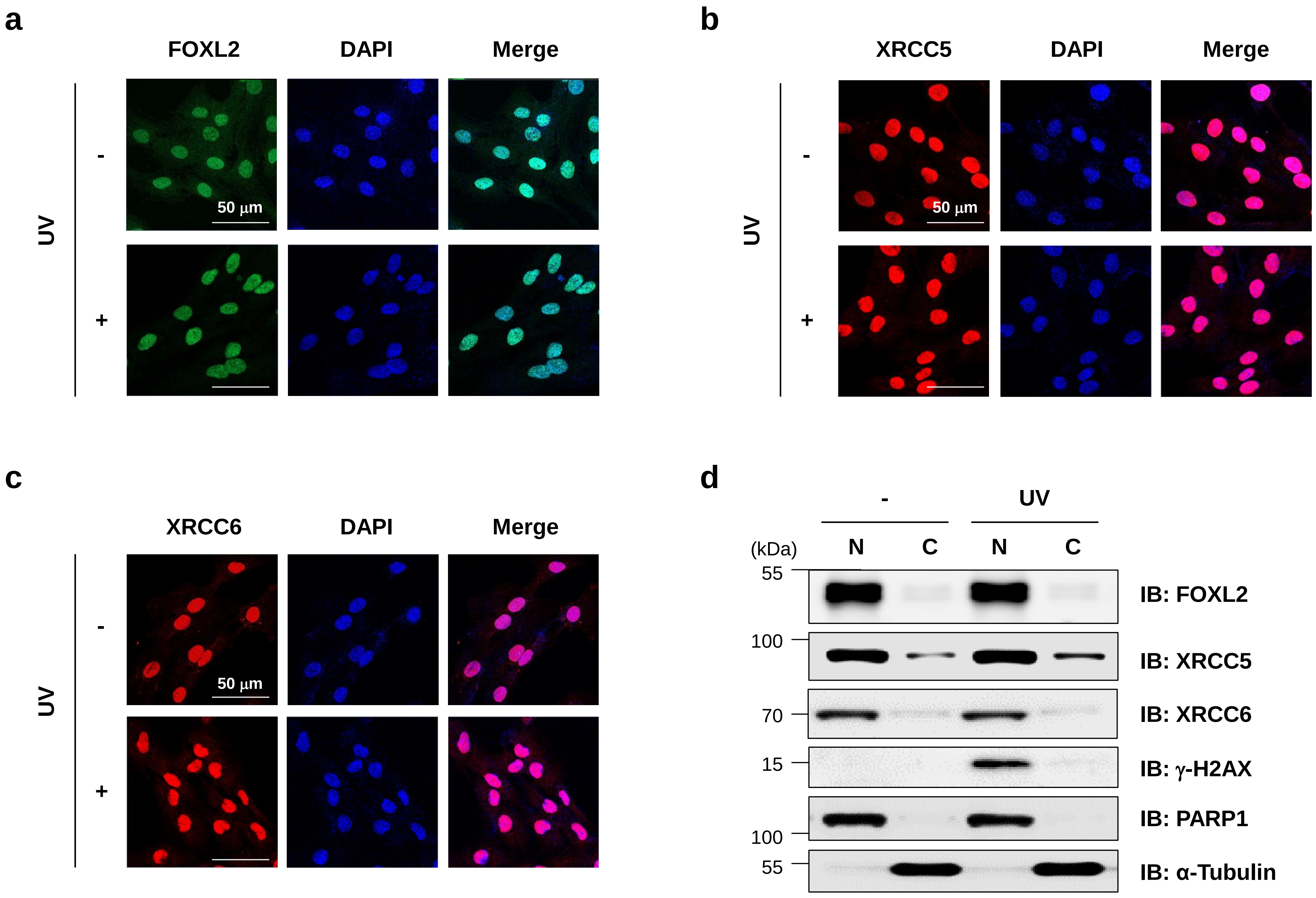

Supplementary Fig. 8. UV does not affect the cellular localization of FOXL2, XRCC5, and XRCC6. a–c After 2 h of UV irradiation, KGN cells were subjected to immunocytochemistry of FOXL2 (**a**), XRCC5 (**b**), and XRCC6 (**c**) (scale bar, 50 μm). (**d**) UV-irradiated KGN cell lysates were fractionated into nuclear (N) and cytosolic (C) fractions, and western blot analyses were performed. PARP1 and α-tubulin were used as controls for the nuclear and cytosolic fraction, respectively. Source data are provided as a Source Data file.

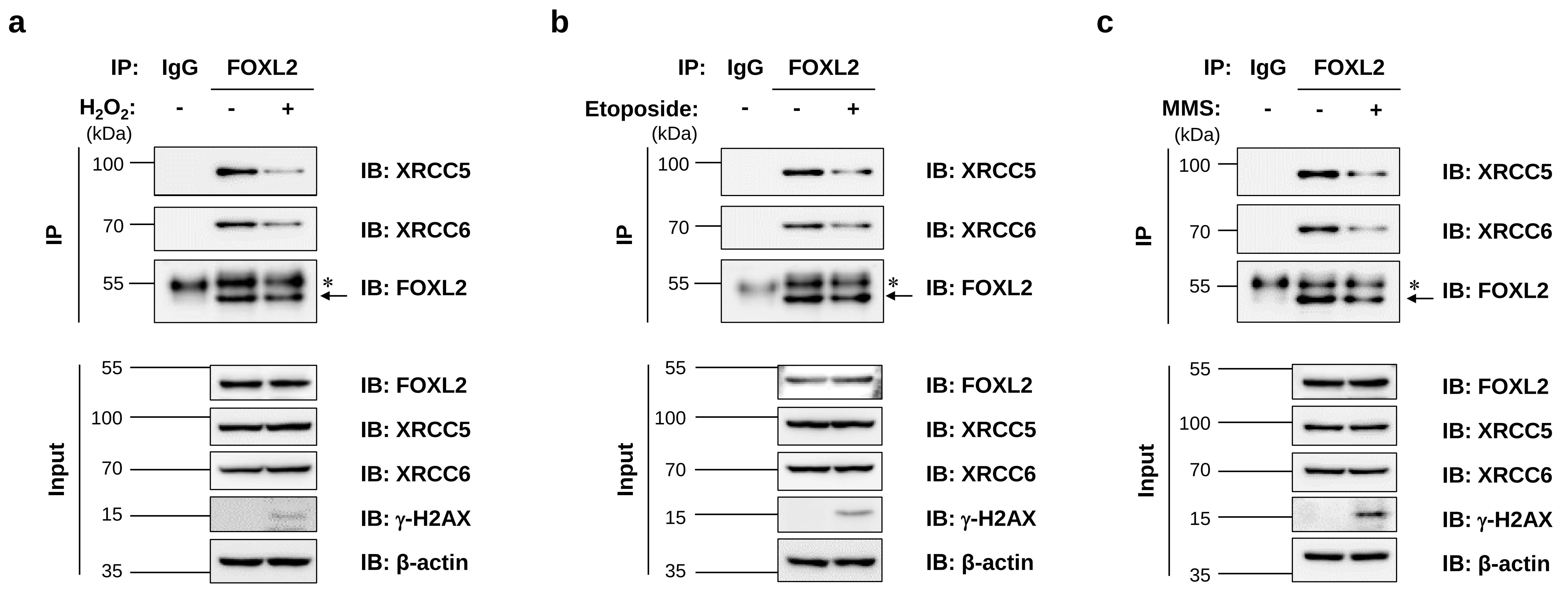

Supplementary Fig. 9. XRCC5/XRCC6 is liberated from FOXL2 in response to DSB stresses. a–**c** H_2O_2 (150 μM, 2 h) (**a**), etoposide (100 μM, 1 h) (**b**), or methyl methanesulfonate (MMS; 1 mM, 0.5 h) (**c**)-treated KGN cells were subjected to XRCC5 and XRCC6 immunoprecipitation with anti-FOXL2 antibody. The amounts of precipitated XRCC5 and XRCC6 were compared with those in untreated cells. Source data are provided as a Source Data file.

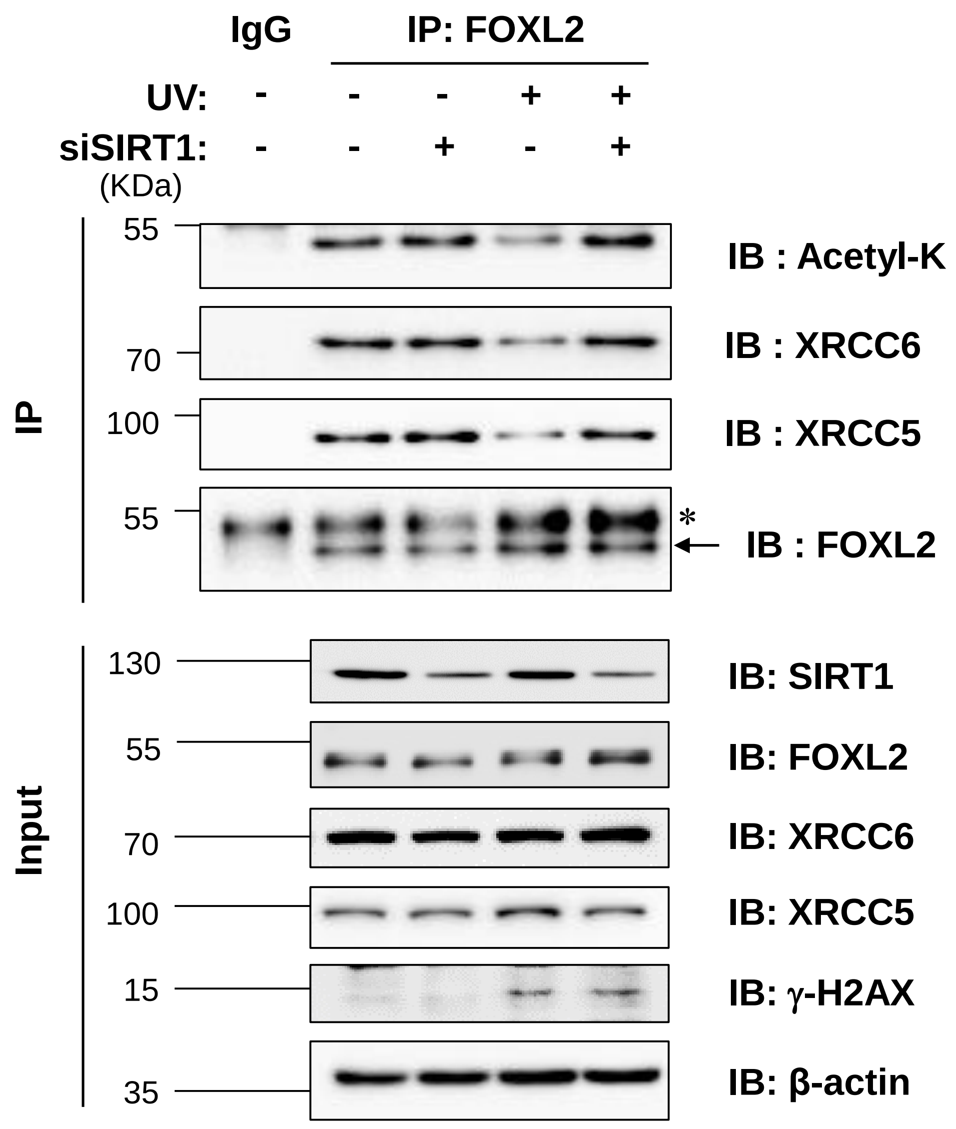

Supplementary Fig. 10. SIRT1 mediates FOXL2 deacetylation. KGN cells were transfected with scrambled or specific siRNAs against SIRT1. After 24 h incubation, the cells were UV-irradiated for 2 h. The cell lysates were immunoprecipitated with anti-FOXL2 antibody following immunoblot analyses using the indicated antibodies. Source data are provided as a Source Data file.

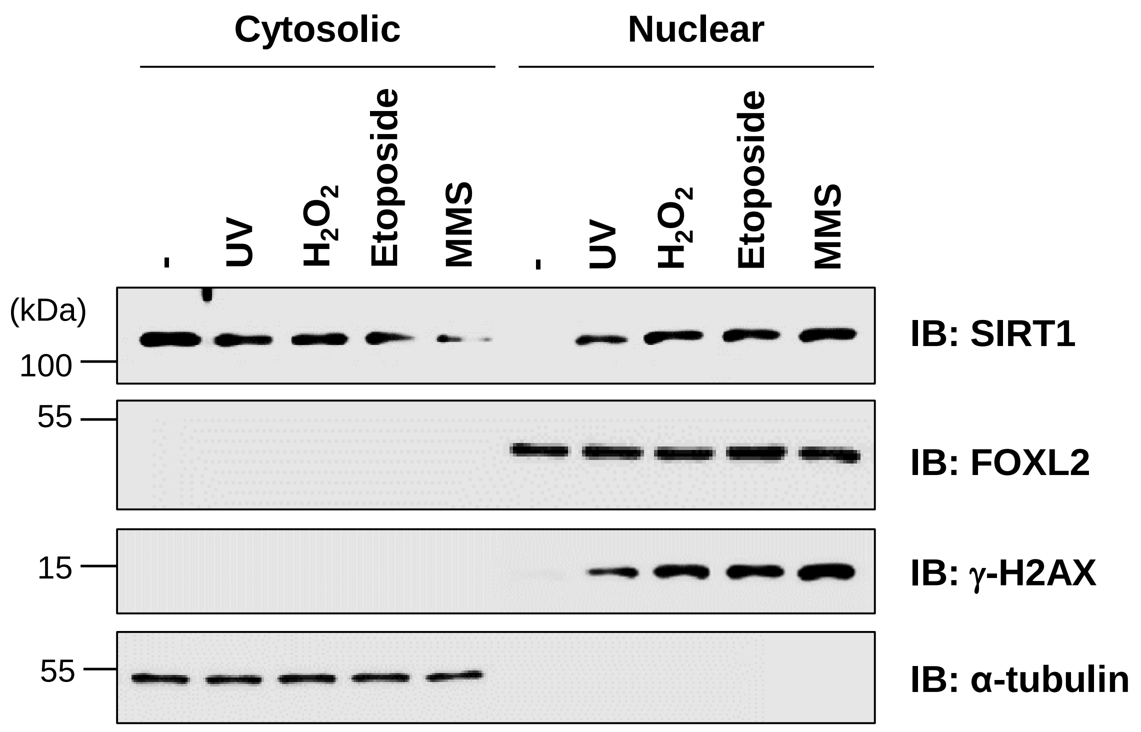

Supplementary Fig. 11. SIRT1 is translocalised from the cytosol to the nucleus following DSB insults in KGN cells. DNA-damaged KGN cell lysates were fractionated into nuclear (N) and cytosolic (C) fractions, and western blot analyses were performed. FOXL2 and α-tubulin were used as controls for the nuclear and cytosolic fraction, respectively. Source data are provided as a Source Data file.

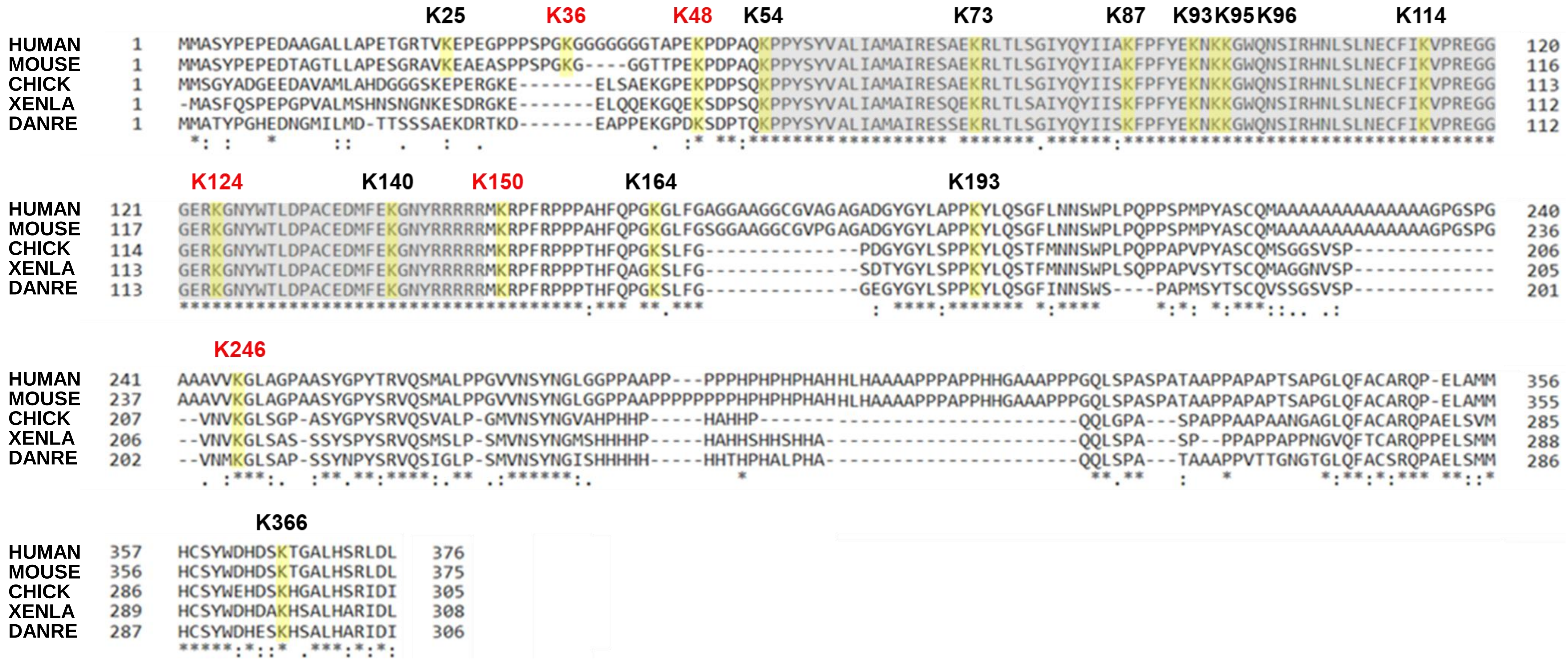

Supplementary Fig. 12. Predicted acetylation sites of FOXL2 lysine residues. Sequence alignment of FOXL2 amino acid sequences from *Homo sapiens* (HUMAN), *Mus musculus* (MOUSE), *Gallus gallus* (CHICK), *Xenopus laevis* (XENLA), and *Danio rerio* (DANRE) using the UniProt alignment tool (http://www.uniprot.org/). The forkhead domain is highlighted in grey, and lysine residues are highlighted in yellow. The five lysine residues of FOXL2 (K36, K48, K124, K150, and K246) that received high predictive scores in Prediction of Acetylation on Internal Lysines (http://bdmpail.biocuckoo.org/prediction.php) and Acetylation Set Enrichment-Based (http://bioinfo.bjmu.edu.cn/huac/predict_p/) are highlighted in red.

Supplementary Fig. 13

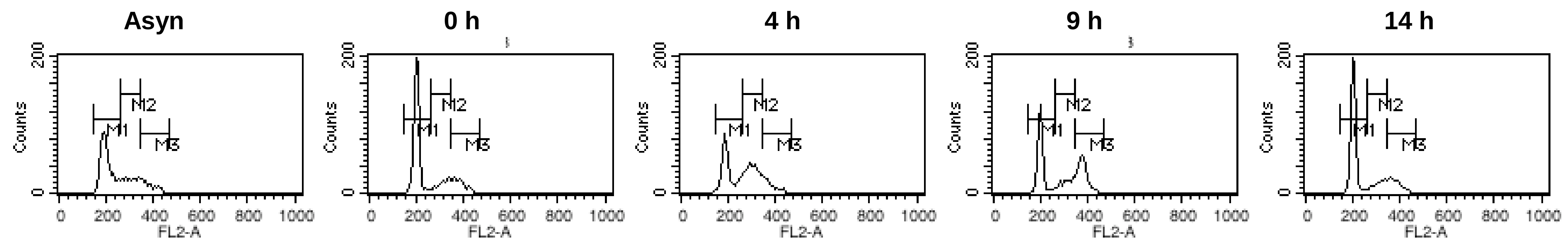

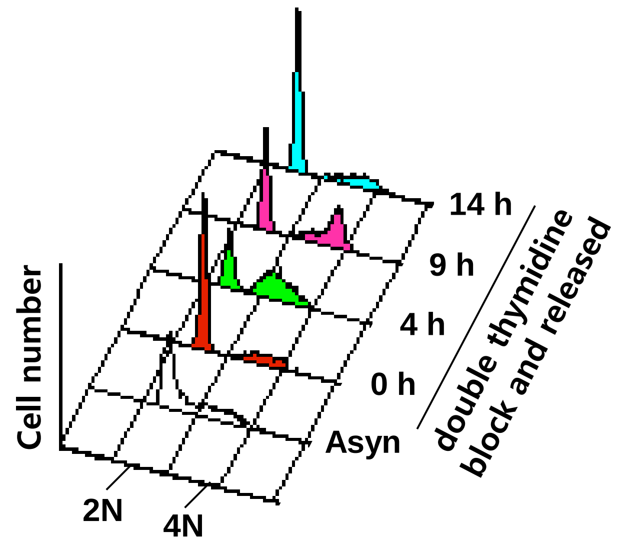

	Asyn	Double thymidine block and released			
		0 h	4 h (S)	9 h (G2)	14 h (G1)
**G1:**	55.16 ± 1.88 %	**71.81 ± 0.18 %**	42.03 ± 0.98 %	46.51 ± 0.38 %	**71.98 ± 0.82 %**
**S:**	25.20 ± 0.58 %	13.98 ± 0.42 %	**42.32 ± 1.50 %**	16.05 ± 1.14 %	15.04 ± 0.35 %
**G2:**	19.64 ± 1.78 %	14.21 ± 0.31 %	15.65 ± 2.40 %	**37.44 ± 0.94 %**	12.98 ± 0.86 %

Supplementary Fig. 13. Cell cycle synchronization. Analysis of cell cycle profiles at indicated time points after release following double thymidine block was performed using flow cytometry. Cells were stained with propidium iodide and analysed based on their DNA content. An asynchronous population of cells was used as a control. Red characters indicate most synchronized cells in S, G2, and G1. Data are the mean ± SEM of three independent experiments performed in triplicates. Source data are provided as a Source Data file.

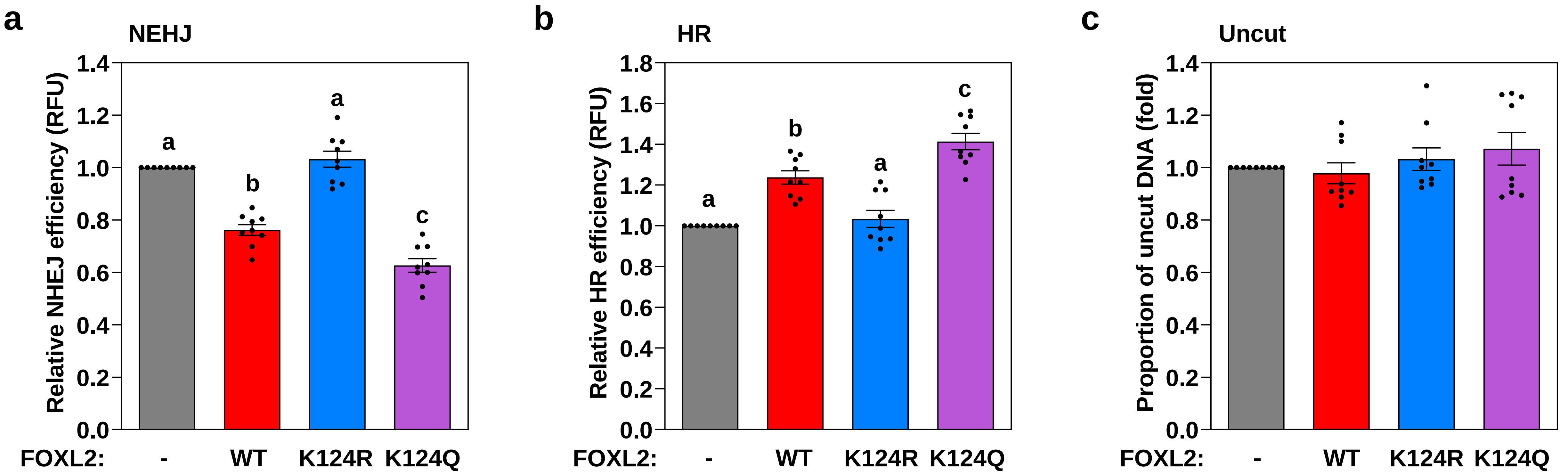

Supplementary Fig. 14. Effect of FOXL2 acetylation mutants on the NHEJ and HR activities. a, **b** The effect of WT, K124R, and K124Q mutants of FOXL2 on NHEJ (**a**) and HR (**b**) activities were assessed in 293TdA3- or 293T-DR-GFP stable cells transfected with an I-*Sce*I endonuclease expression plasmid and indicated as relative fluorescence unit. **c** 293TdA3-stable cells expressing I-*Sce*I were transfected with plasmid expressing empty vector (control), FOXL2, K124R, or K124Q mutants. After 48 h, the proportion of uncut DNA in the cells was measured by quantitative PCR. Data are the mean ± SEM of three independent experiments performed in triplicate. Different letters denote statistical significance (Student–Newman–Keuls test; $p < 0.05$). Source data are provided as a Source Data file.

Supplementary Fig. 15

a

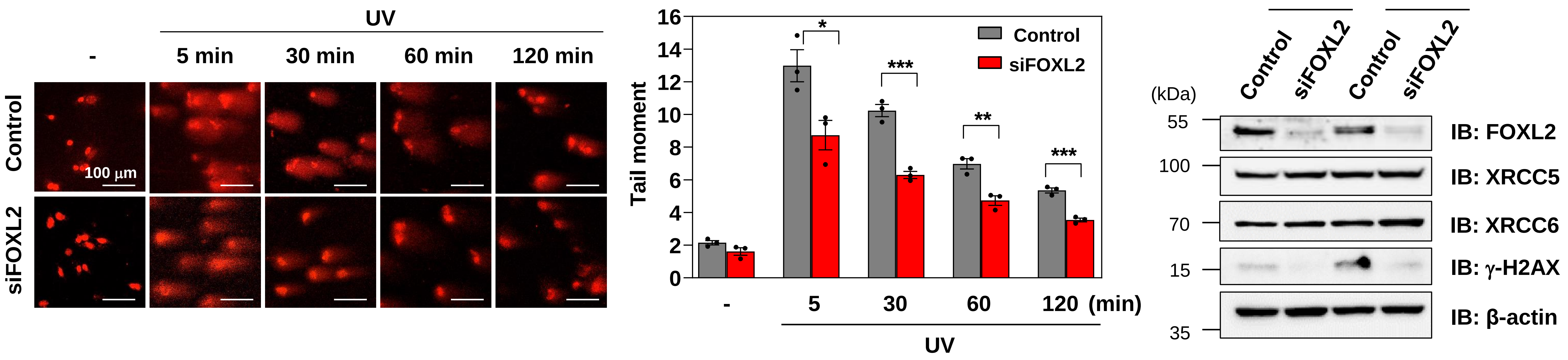

b

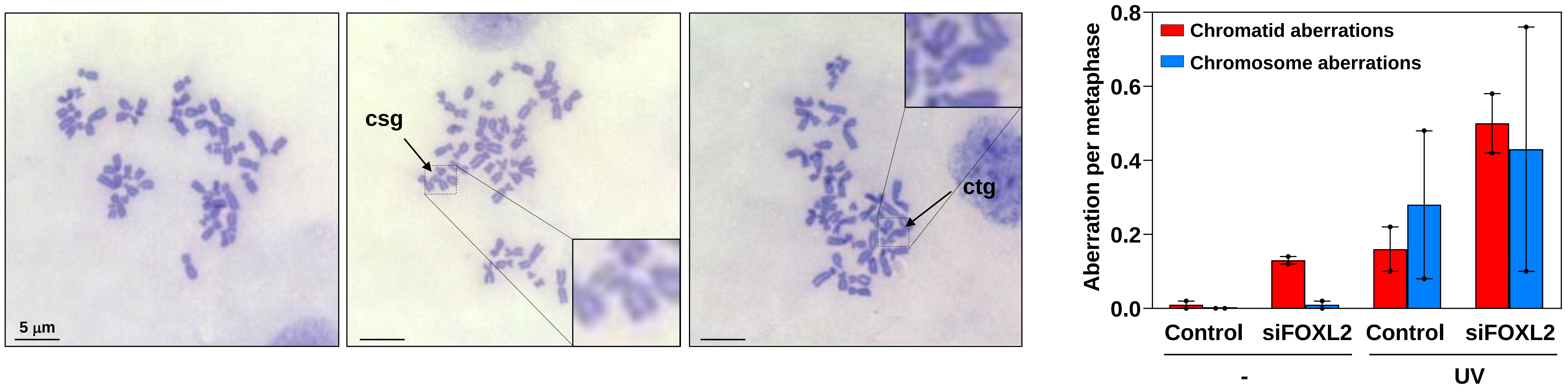

UV	Cell types	Total cells scored	Normal	Chromatid aberrations					Chromosome aberrations					Mitotic index
				Chromatid gaps	Chromatid breaks	Iso chromatid gaps	Chromatid exchange	Total chromatid	Dicentrics	Rings	Excess acentics	Tricentrics	Total chromosomal	
-	**Control**	**100**	**99**	**0**	**1**	**0**	**0**	**1**	**0**	**0**	**0**	**0**	**0**	**2.8**
	**siFOXL2**	**100**	**86**	**4**	**7**	**0**	**2**	**13**	**0**	**0**	**1**	**0**	**1**	**3.6**
+	**Control**	**100**	**65**	**4**	**9**	**3**	**0**	**16**	**17**	**1**	**10**	**0**	**28**	**1.5**
	**siFOXL2**	**100**	**35**	**20**	**20**	**6**	**4**	**50**	**25**	**1**	**16**	**1**	**43**	**2.5**

Supplementary Fig. 15. FOXL2 is required for maintaining genome stability. a A neutral comet assay was performed and analysed as described in the legend of Fig. 6a, except that FOXL2-knockdown KGN cells were used in this experiment. Data are presented as the mean ± SEM of three independent experiments. The *p* values were analysed by unpaired, two-tailed Student's *t*-test (**p* < 0.05, ***p* < 0.01, ****p* < 0.001). **b** Chromosomal aberration analysis was conducted, and the data were analysed as described in the legend of Fig. 6b, except that FOXL2-knockdown KGN cells were used instead of FOXL2 KO cells. Representative Giemsa-stained metaphases from the cells (left upper) and quantified results (right upper) are shown. The length of scale bar is 5 μm. Numerical data are provided in the table (lower panel). Aberrations are indicated by arrows; csg, isochromatid gap; ctg, chromatid gap. Source data are provided as a Source Data file.

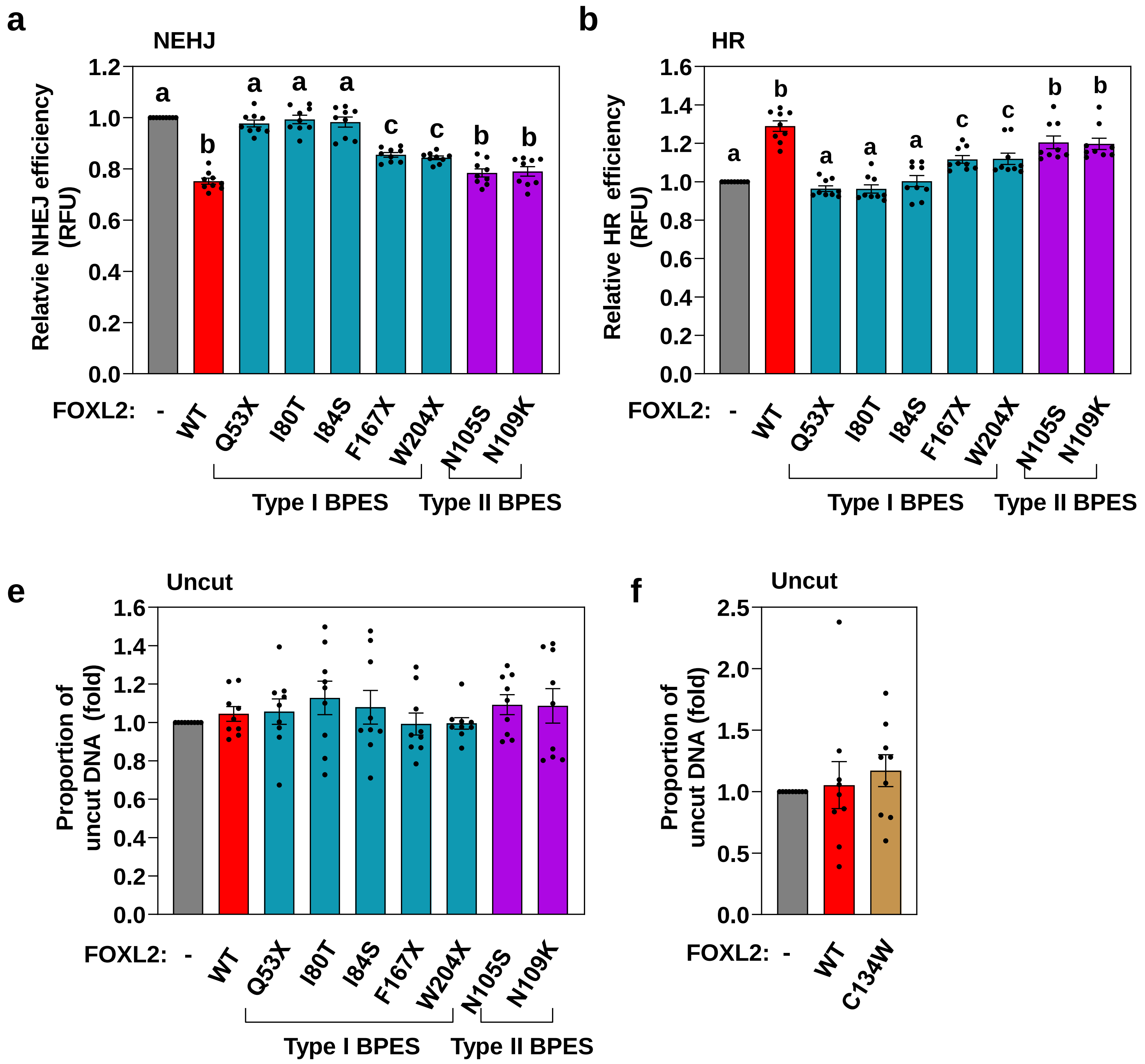

Supplementary Fig. 16. Assessment of the NHEJ and HR activities of BPES- and AGCT-associated FOXL2 mutants. a–**f** 293TdA3- or 293T-DR-GFP stable cells expressing I-*Sce*I were transfected with plasmid expressing empty vector (control), pCMV-Myc-FOXL2, or BPES-associated FOXL2 mutants, including Q53X, I80T, I84S, F167X, W204X, N105S, and N109K (**a**, **b**, and **e**), or AGCT-associated C134W FOXL2 mutant (**c**, **d**, and **f**). After 48 h, the relative NHEJ (**a** and **c**) and HR (**b** and **d**) efficiencies were presented as relative fluorescence unit. BPES- (**e**) or AGCT-associated (**f**) FOXL2 mutants on the proportion of uncut DNA in the cells was measured by quantitative PCR. Data are the mean ± SEM of three independent experiments performed in triplicate. Different letters denote statistical significance (Student–Newman–Keuls test; $p < 0.05$). Source data are provided as a Source Data file.

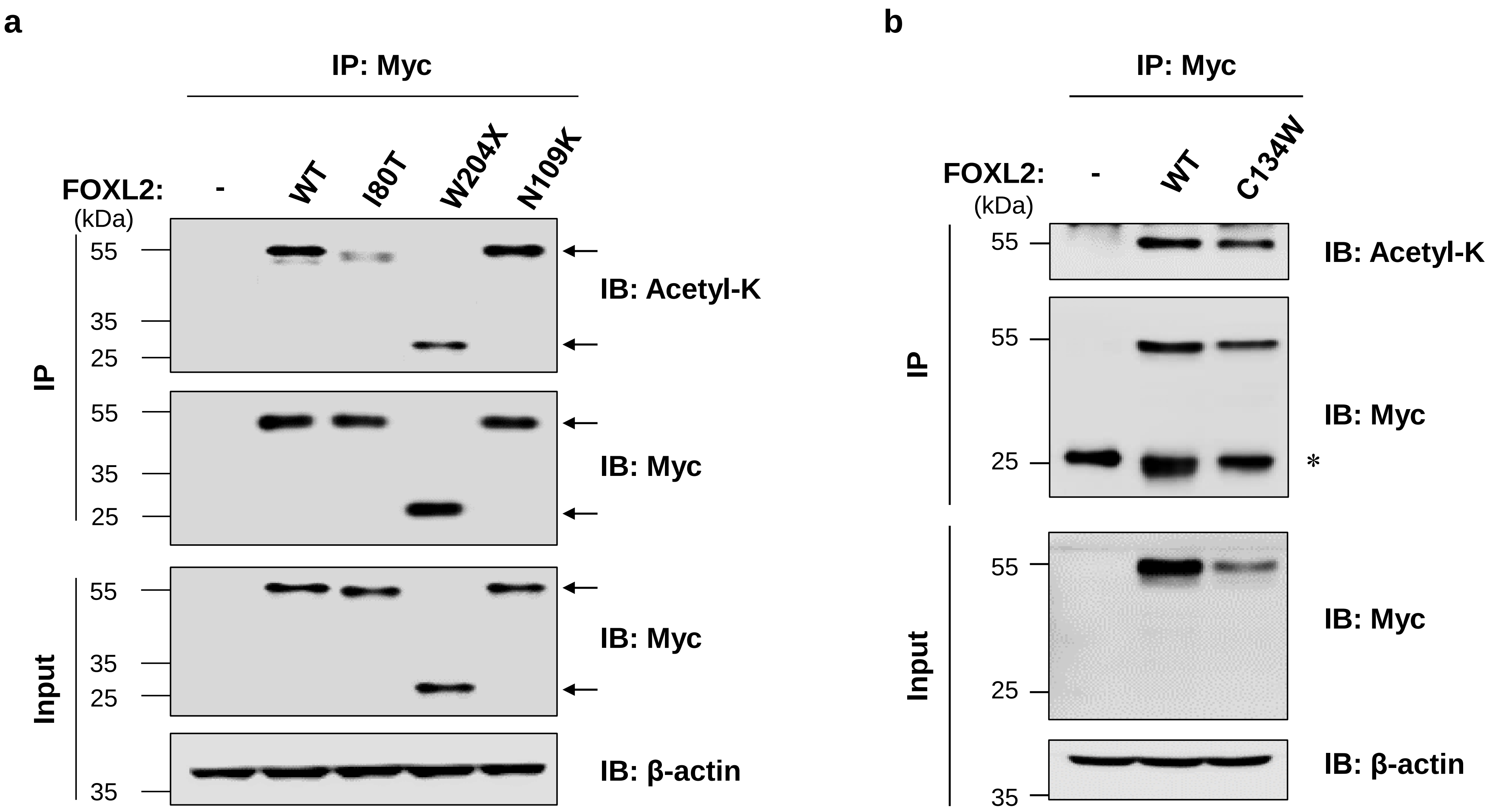

Supplementary Fig. 17 Changes of FOXL2 Lysine Acetylation by Pathogenic FOXL2 Mutants

a, b KGN cells were transfected with pCMV-Myc empty vector, FOXL2 WT, I80T, W204X, N109K (**a**), or C134W (**b**). The lysates were immunoprecipitated with anti-Myc antibody and immunoblotted with indicated antibodies. Source data are provided as a Source Data file.

Supplementary Fig. 18

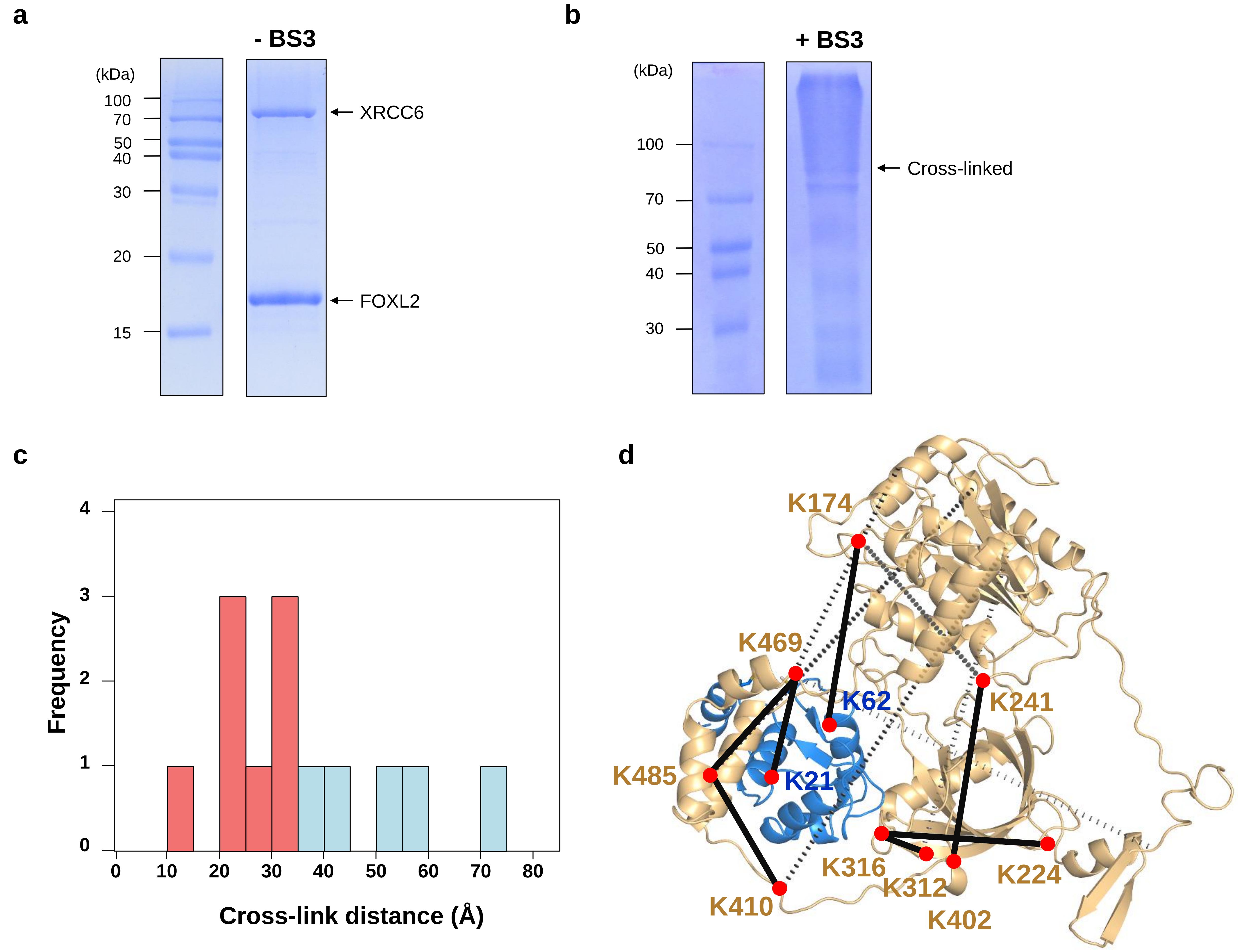

e

```
HUMAN    1   ------------------MSGWESYYKTEGDEEAE---EEQEENLEASGDYKYSGRDSLIFLVDASKAMFESQS-EDELTPFDMSIQCIQSVYISKIISSDRDLLAVVFYGTEKDKNS-V   97
MOUSE    1   ------------------MSEWESYYKTEGEEEEE---E--EESPDTGGEYKYSGRDSLIFLVDASRAMFESQG-EDELTPFDMSIQCIQSVYTSKIISSDRDLLAVVFYGTEKDKNS-V   95
CHICK    1   MEMWVLGEVGMAVLSAAAMADWVSYYRGDGPDEEEDGEQQEEEGPEAVADYRFSGRDSLIFLVDASKAMFEPYENEEAATPFDMTMQCIRNVYTSKIISSDKDLLSVVFYGMENNKNS-A   119
XENLA    1   ------------------MTEWGDHFVNQEVEDD----EEQEEGESDASAFRFSGRDSLIFLVDASKPMFESID--GELTPFDLTLQCIRSVYASKIISSDHDLLSVVFFGTRESTNC-D   95
DANRE    1   ------------------MANWGNYFPNEDDVDEQ-------DEEESGGDYKSSGRDSLVFLVDASKEMFIKGE-NGEPSNFDMTMQCVRSVYTSKIISSDRDLVALVFYGTEQSKNPRN   94
                                *: * .::  :   :          :  .  . :: ******:******: **         : **:::**::.** *******:**:::**:* .:..*

HUMAN    98  NFKNIYVLQELDNPGAKRILELDQFKGQQGQKRFQDMMGHGSDYSLSEVLWVCANLFSDVQFKMSHKRIMLFTNEDNPHGNDSAKASRARTKAGDLRDTGIFLDLMHLKKPGGFDISLFY   217
MOUSE    96  NFKNIYVLQDLDNPGAKRVLELDQFKGQQGKKHFRDTVGHGSDYSLSEVLWVCANLFSDVQLKMSHKRIMLFTNEDDPHGRDSAKASRARTKASDLRDTGIFLDLMHLKKPGGFDVSVFY   215
CHICK    120 DFKHIYVLQELDNPGAKRILELDQYRGDEGRVLFRETFGHNADYSLGEALWACSNLFSDVRVRLSHKRIMLFTNEDNPHANDSAKAKLARTRAGDLRDTGIILDLMHLKKPGGFDISLFY   239
XENLA    96  PFKHLCVLHDLDTPGAKRILDLDKYKEEKGRALFCDTIGCGGDFSLGEALWLCSNLFSNVKVKMSHKRIMLFTNEDNPHANDPAKAKQARAKAEDLKDMGIFLDLMHLEKPGGFDISLFY   215
DANRE    95  SFKHVYVYHDLESPGAKRVQDIDKLKGDKGGQFAEKTMGSG-ETSLGEALWCCSNLYSDIKLRLSHKRLMIFTCRDEPHGGDSAKDRQARTKAADLKETGVAIDLMHLSKPGGFDVSLFF   213
              **:: * ::*:.*****: ::*: : ::*     . .* . : **.*.** *:**:*:::.::****:*:** .*:**. * **   **::* **:: *: :*****.******:*:*:

HUMAN    218 RDIISIAEDE-DLRVHFEESSKLEDLLRKVRAKETRKRALSRLKLKLNKDIVISVGIYNLVQKALKPPPIKLYRETNEPVKTKTRTFNTSTGGLLLPSDTKRSQIYGSRQIILEKEETEE   336
MOUSE    216 RDIITTAEDE-DLGVHFEESSKLEDLLRKVRAKETKKRVLSRLKFKLGEDVVLMVGIYNLVQKANKPFPVRLYRETNEPVKTKTRTFNVNTGSLLLPSDTKRSLTYGTRQIVLEKEETEE   334
CHICK    240 RDIINVAEDE-DLGIQPDESGKLEHLMKKVRAKETRKRALSRLNLYLNKDLSFSVGVYNLIQKAYKPYPVKLYRETNEPVKTKTRVFNGKTGSLLLPSDTKRAQTYGNRQIAMEKEETEE   358
XENLA    216 RDIVNTAEDE-DLVVQFKASEKLDDLLKKVRAKEAKKRALSRLNLKLGPDVGLTVGVYNLVQKAVKPPTVRLYRESNEPVKTKTRIFHSNTGSLLLPSDTKRSQTYGNRQIVLEKDETEQ   334
DANRE    214 CDIVSPPEDESELGLQIEPCRKLEDLQKRVRAKELKKRAQCRLTFSLGEGVDLAVGVYVLARTAMKPSAVKLYRDNNEPVRTKSRLFHTQTGGILLPNDTKRAQVYGQKQIVMEKDEVDE   333
              **:.  *** :* :: . . **:.* ::***** :**. .**.: *. .: : **:* * :.* **  ::***:.****:**:* *: .**.:***.****:  ** :** :**:*.::

HUMAN    337 LKRFDDPGLMLMGFKPLVLLKKHHYLRPSLFVYPEESLVIGSSTLFSALLIKCLEKEVAALCRYTPRRNIPPYFVALVPQEEELDDQKIQVTPPGFQLVFLPFADDKRKMPFTEKIMATP   456
MOUSE    335 LKRFDEPGLILMGFKPTVMLKKQHYLRPSLFVYPEESLVSGSSTLFSALLTKCVEKKVIAVCRYTPRKNVSPYFVALVPQEEELDDQNIQVTPGGFQLVFLPYADDKRKVPFTEKVTANQ   454
CHICK    359 VKRFDSPGLFLIGFKPLSMLKQHHHIRPSQFMYPEESLVTGSTTLFNALLMKCLEKEVMALCRYIARRNTPPRIVALIPQEEEVDEQKVQIAPPGFHIIFLPYADDKRNVDFTEKVPANR   478
XENLA    335 LKRFDEPGLVLIGFKPISCLKKHHFTRPAQFIYPEESIITGSTTLFNALLTRCLARQVMAICRYTPRRNTPPRFVALVPQDEELDDQNMQSAPSGFNLVCLPFADDIRKLDTPEKITANE   454
DANRE    334 IKKFDDPGLVLIGFKPIDRLKLHHHLRPALFIYPEEEQISGSSCMFTALLLKCCEKNVFALCKYIPRRNTPPRFVALVPQREELDQSQTQATPPGFHVIYLPFADDIRTVDPHVGPTASD   453
             :*:**.***.*:****    ** :*. **: *:****. : **: :*.*** :*   ::* *:*:*   *:*   * :***:** **:*:.: * :* **::: **:*** *.:       *.

HUMAN    457 EQVGKMKAIVEKLRFTYRSDSFENPVLQQHFRNLEALALDLMEPEQAVDLTLPKVEAMNKRLGSLVDEFKELVYPPDYNPEGKVTKRKHDNEGSG----SKRPKVEYSEEELKTHISKGT   572
MOUSE    455 EQIDKMKAIVQKLRFTYRSDSFENPVLQQHFRNLEALALDMMESEQVVDLTLPKVEAIKKRLGSLADEFKELVYPPGYNPEGKVAKRKQDDEGST----SKKPKVELSEEELKAHFRKGT   570
CHICK    479 EQVDKMKGIIQKLRFKYRTDSFENPVLQQHFRNLEALALDMLEPEQAEDLTMPKTEEMSRRLGNLVEEFKQLVYPPDYSPEGKAAKRKQAGDAQA----EKRPKIEISEDSLRSYVQNGT   594
XENLA    455 EQVDKMKEIVHKLRFNFRSDSFENPVLQQHFRNLEALALDMLEPEPIEDLTLPKVEMIDRRIGSLAEEFKELVYPPGYNPEGKAVKRKQGGSSDSRVEKKAKADVSISEDELRTYVKNGT   574
DANRE    454 EQVDKMKEIVHKLRFKYRSDAFENPVLQQHYSNLEALALDMLSPEPIEDLTMPKVKMMDDRLGPLVQEFKDLVYPRDYNPEGKPAAKRKPAESGG-GAAEKKAKVEISEEELKIHVAKGT   572
             **:.*** *:.****.:*:*:*********: ********::. *   ***:**.: :. *:* *.:***:****  .*.****  . :::  ..       . : .:. **:.*: :. :**

HUMAN    573 LGKFTVPMLKEACRAYGLKS-GLKKQELLEALTKHFQD-   609
MOUSE    571 LGKLTVPTLKDICKAHGLKS-GPKKQELLDALIRHLEKN   608
CHICK    595 LGKLTVSALKDTCRHYGLRS-GGKKQELIDALTEYFSGR   632
XENLA    575 LGKLTVPALKEACRGYKLK--GSKKQELVDALVEYFKKN   611
DANRE    573 LGKLTVPVLKDACKQFNIRTTGTKKQELIDALTMQLSK-   610
             ***:**  **: *: . ::  * *****::**    :.
```

Supplementary Fig. 18. XL-MS Analysis of FOXL2 and XRCC6

a Purified FOXL2-FH (K124Q) and XRCC6 were mixed with 1:1 molar ratio and analysed by SDS-PAGE. **b** BS3-crosslinked complex analysed by SDS-PAGE is shown. **c** The histogram is showing the Cα–Cα distances of all identified cross-linked peptides. Cα-Cα distances below 35 Å, which is an appropriate distance for crosslinks, are highlighted in light red. **d** Cross-linked lysine residues indicated with red dot are shown in our proposed docking model, which suggest that the integrative docking model is consistent with the XL-MS data. Cα-Cα distances below 35 Å are shown in thick black line, and those above 35 Å are shown in dashed gray line. **e** Sequence alignment of XRCC6 amino acid sequences from *H. sapiens* (HUMAN), *M. musculus* (MOUSE), *G. gallus* (CHICK), *X. laevis* (XENLA), and *D. rerio* (DANRE) using the UniProt alignment tool (http://www.uniprot.org/). The Ku core domain is highlighted in gray. Source data are provided as a Source Data file.
